# Induced Abortion After Previous Caesarean Section: A Scoping Review

**DOI:** 10.1111/ajo.70013

**Published:** 2025-04-11

**Authors:** Natalie Drever, Vinay Gangathimmaiah, Brittany van Der Lugt, Cecelia O'Brien, Catriona Melville, Kirsten Black, Caroline de Costa

**Affiliations:** ^1^ College of Medicine and Dentistry James Cook University Cairns Queensland Australia; ^2^ Department of Obstetrics and Gynaecology Cairns Hospital Cairns Queensland Australia; ^3^ College of Medicine and Dentistry James Cook University Townsville Queensland Australia; ^4^ Department of Emergency Medicine Townsville University Hospital Townsville Queensland Australia; ^5^ Department of Obstetrics and Gynaecology Townsville University Hospital Townsville Queensland Australia; ^6^ Department of Obstetrics and Gynaecology Logan Hospital, Metro South Hospital and Health Service Meadowbrook Queensland Australia; ^7^ Department of Obstetrics, Gynaecology and Neonatology, School of Medicine University of Sydney Sydney New South Wales Australia; ^8^ The Cairns Institute James Cook University Cairns Queensland Australia

**Keywords:** abortion, accreta, caesarean section, review, termination of pregnancy

## Abstract

**Background:**

Previous caesarean section (CS) is increasingly common among women undergoing induced abortion.

**Aims:**

To map and analyse existing literature on abortion safety, outcomes and management in those with previous CS.

**Materials and Methods:**

Four databases were systematically searched from inception to July 2024. Primary human studies in English reporting on outcomes, safety or management of first‐ or second‐trimester medical (MToP) or surgical (SToP) abortion in women with previous CS were included. Uterine rupture incidence was analysed cumulatively in the first and secondtrimesters by the number of CS and the type of prostaglandin used. Data on the efficacy and safety of MToP and SToP, including studies reporting on the management of abortion in the setting of abnormal placentation, were collected and analysed by theme.

**Results:**

In total, 164 articles met inclusion criteria. Incidence of uterine rupture in first‐trimester MToP was 0 of 2194 cases, in second‐trimester misoprostol MToP in those with 1 previous CS was 0.5% (10/1910) and 2.2% (18/835) in women with ≥ 2 CS (*p* < 0.001). Mifepristone priming did not increase the rupture rate in second‐trimester MToP (*p* = 0.77). Previous CS was a modest risk factor for retained products after MToP across both trimesters (OR 1.48, CI 1.29–1.70).

**Conclusion:**

Medical and surgical abortion in the first and second trimester appears safe in women with prior CS; however, risks include uterine rupture, need for surgical intervention and haemorrhage from undiagnosed placenta accreta. Further research and guidance are needed on managing abortion after previous classical CS, ≥ 3 previous CS and those with abnormally invasive placenta.

## 1 Background

Caesarean section (CS) rates continue to rise internationally [1], and in Australia climbed from 32% to 38% between 2009 and 2021 [2, 3]. Furthermore, the majority (88%) of Australian women who have a CS will have their subsequent birth by CS [3], leading to an overall increasing trend in women with more than one previous CS. There are at least 80,000–90,000 induced abortions per year in Australia, and approximately a quarter of pregnancies end in induced abortion [4]. Thus, it is common for an individual undergoing induced abortion to have had one or more CS. CS carries specific risks to future pregnancies, including abnormal placentation, caesarean scar pregnancy (CSP) and uterine rupture, which also have the potential to affect abortion safety [5, 6].

The incidence of uterine rupture during second‐trimester MToP has been reported to be lower than in term vaginal birth in those with previous CS [7] (0.3%–0.43% in previous systematic reviews) [6, 8], a recent meta‐analysis published in 2023 reports a 1.1% incidence with mifepristone‐misoprostol [9]. Current guidelines acknowledge the small risk of rupture with second‐trimester MToP, some offering consensus‐based low‐dose misoprostol regimens for women with previous CS [10, 11, 12]. These regimens are heterogeneous between institutions, and there is little guidance regarding SToP safety and optimal abortion care for those with > 1 previous CS or previous vertical uterine incision (classical CS). Importantly, previous reviews have included low numbers of individuals with > 1 previous CS, making it difficult to accurately draw conclusions about rupture rates in this group.

With rising CS rates, related sequelae including placenta accreta spectrum (PAS) and CSP are increasingly reported [13, 14]. Abnormal placentation, with associated obstetric risks, can also be a reason for seeking abortion; and there is minimal guidance on optimising the safety of abortion in these cases.

Given the scope of the research questions and heterogenous nature of available evidence, the exploratory approach of a scoping review was chosen [15]. This scoping review covers a broader topic than previously published reviews and includes both first and second‐trimester abortion and varying methods of medical and surgical abortion.

## 2 Materials and Methods

### 2.1 Protocol and Registration

This review was performed according to Joanna Briggs Institute methodology [16] using the Preferred Reporting Items for Systematic Reviews and Meta‐Analyses Extension for Scoping Reviews (PRISMA‐ScR) [17] (Table S1). The protocol was registered with Open Science Framework (OSF.IO/AH79V).

### 2.2 Identification of Research Questions

The objectives of the review were as follows:

- To summarise and analyse existing literature on abortion after prior CS, including risks of complications.
- To map the current evidence on recommended management of abortion after CS.
- To identify knowledge gaps and guide further research into abortion care for those with previous CS.

### 2.3 Search Strategy

We systematically searched four online databases (MEDLINE (Ovid), CINAHL, SCOPUS and EMBASE) from inception to July 2024. The search strategy was developed using keywords and MeSH terms related to outcomes, complications and management of abortion after prior CS (Appendix S1). References of included articles were hand‐searched to identify additional relevant articles.

### 2.4 Study Selection

Title and abstract screening were performed independently by three reviewers (ND, VG and BV), followed by full‐text review (ND and VG) to determine eligibility. Conflicts between the two initial reviewers were resolved by a third (CD).

Articles meeting eligibility criteria were included (Table S2). Studies were included if they reported on outcomes (safety; complete abortion rates; complication rates, prevention or management) of first or second‐trimester abortion in individuals with ≥ 1 previous CS. Studies only involving participants with miscarriage or intrauterine fetal death, and those that did not report outcomes for participants with previous CS, were excluded. Studies involving women undergoing treatment of known CSP were excluded, as this would have yielded papers regarding management of this condition, outside the scope of this review. However, studies and case reports of women undergoing abortion with undiagnosed CSP that became apparent after commencement of the abortion process were included, as this is an increasingly common challenge facing abortion care clinicians. Studies were also included if they involved participants undergoing second‐trimester abortion with PAS and previous CS.

Inclusion was limited to articles published in English, involving humans, with no date limitations. Primary descriptive, observational, and interventional studies were included, as were case reports. Secondary sources of evidence including systematic reviews, opinions, book chapters, letters to the editor, protocols and guidelines were excluded.

### 2.5 Data Extraction and Analysis

Data variables are summarised in Table S3. Risk of bias was assessed by two reviewers using the Cochrane Risk of Bias for Randomized Trials Tool version 2.0 (RoB 2.0) [18] for randomised controlled trials, ROBINS‐I [19] for non‐randomised interventional studies and ROBINS‐E [20] for observational studies.

Results were grouped and analysed according to themes:

- Safety and efficacy of MToP in first‐trimester
- Safety and efficacy of MToP in second‐trimester
- Safety and outcomes of SToP in first‐ and second‐trimester
- Abortion after previous classical CS
- Abortion in the context of abnormal placentation

Descriptive statistics were used and cumulative meta‐analyses of rupture rates in the setting of first‐ and second‐trimester MToPs, and for cervical priming prior to SToP, were performed. Confidence intervals (CI) were calculated using the adjusted Wald method [21]. Data analysis of the efficacy of first‐ and second‐trimester MToP, and outcomes of first‐ and second‐trimester SToP, was performed using Review Manager (Revman) 5.4.1 with risk ratios and CI given using a random effects model. Results were considered statistically significant when *p* < 0.05.

### 2.6 Definitions

First trimester is defined as < 13 weeks gestation and second trimester 13–28 weeks; however, several studies included in the analysis defined the second‐trimester as beginning at 12 + 0, and these were included in the second‐trimester analysis if these data were unable to be extracted separately. Classical CS refers to a vertical incision on the uterus, as opposed to lower segment CS, in which a transverse lower segment incision is made. Hysterotomy refers to operative abdominal delivery with uterine incision for a non‐viable pregnancy.

### 2.7 Patient and Public Involvement

Patients and the public were not involved in the design or conduct of this scoping review.

### 2.8 Ethics Statement

Not applicable.

## 3 Results

**FIGURE 1:**
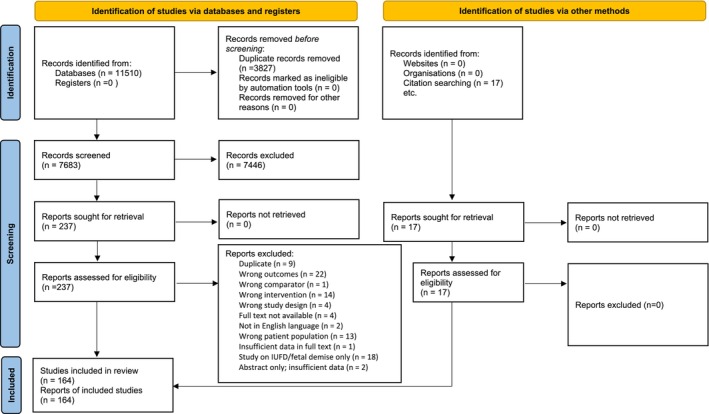
PRISMA flow diagram. *Source:* Page et al. [22]. For more information, visit: http://www.prisma‐statement.org/.

In total, 164 articles were included: 46 case reports in 39 articles (Table S4), and 125 original articles (Table S5). Figure 1 shows the PRISMA diagram of study inclusion.

Table 1 summarises the characteristics of included studies.

**TABLE 1:** Summary of characteristics of included studies.

Characteristics	Values	Number (%) of articles
Year of publication	1981–1990	1 (0.6%)
	1991–2000	11 (6.7%)
	2001–2010	52 (31.7%)
	2011–2020	69 (42.1%)
Continent of conduct	2021–2024	31 (18.9%)
	Africa	12 (7.3%)
	Asia	65 (40.0%)
	Europe	43 (26.2%)
	North America	36 (21.9%)
	Oceania	8 (4.9%)
Design	Case reports	39 (23.8%)
	Case series/descriptive	12 (7.3%)
	Case–control	2 (1.2%)
	Cohort	95 (57.9%)
	Non‐randomised controlled	1 (0.6%)
	Randomised controlled	15 (9.1%)
Gestation	First trimester (< 13 weeks)	24 (14.6%)
	Second trimester (13 + 0–28 + 0 weeks)	121 (73.8%)
	Both	18 (11.0%)
Type of abortion	Unspecified	1 (0.6%)
	MToP	118 (72.0%)
	SToP	39 (23.8%)
	Both	7 (4.3%)

### 3.1 Safety of MToP After Previous Caesarean

There were 23 case reports of uterine rupture complicating either MToP or during cervical ripening before SToP [23, 24, 25, 26, 27, 28, 29, 30, 31, 32, 33, 34, 35, 36, 37, 38, 39, 40, 41]. Twenty (87.0%) cases occurred in the second trimester, and reported blood loss was 200–3000 mL; 4(17.4%) required hysterectomy. Laparotomy was the most common method of surgical intervention for uterine rupture, but four authors described a laparoscopic approach and one reported transvaginal approximation of the defect [27, 32, 34, 39, 41].

Despite three reports of uterine rupture in the first trimester [24, 33, 40], seven observational studies reported no cases of rupture amongst 2194 women undergoing first‐trimester MToP [42, 43, 44, 45, 46, 47, 48] (Table 2).

**TABLE 2:** Incidence of first‐trimester rupture during MToP in included studies.

Authors/publication date	# participants with previous CS	Method of MToP	Gestation	1 CS	≥ 2CS	≥ 3CS	Type of previous CS	Ruptures
Au et al. [48] (2024)	140^a^	600 mg mifepristone followed by 600 μg misoprostol	< 13 weeks	75	65		Unspecified	0
Chien et al. [42] (2009)	122	600 mg mifepristone followed by PO misoprostol 48 h later	< 8 weeks	60	62		Unspecified	0
Gao et al. [43] (1999)	213	150 mg mifepristone in divided doses followed by 600 μg PO misoprostol day 3	4–9 weeks	N/A	N/A	N/A	Unspecified	0
Gautam et al. [44] (2003)	66	50 mg IM MTX followed by 800 μg PV misoprostol 2–3 days later	< 9 weeks	46	20		LSCS	0
Wang et al. [45] (2010)	668	150 mg mifepristone in divided doses followed by 600 μg PO misoprostol day 3	< 7 weeks	589	79		LSCS	0
Xu et al. [46] (2001)	35	150 mg mifepristone in divided doses followed by 600 μg PO misoprostol day 4	< 49 days	35	0	0	Unspecified	0
Young et al. [47] (2022)	950	200 mg PO mifepristone followed by 800 μg PV or SL misoprostol	< 10 weeks	N/A	N/A	N/A	Unspecified	0
Totals (%)	2194			805	226			0 (0%)
[95% CI]								[0.00–0.00]

Abbreviations: CS, caesarean section; LSCS, lower segment caesarean section; MToP, medical termination of pregnancy; N/A, not available.

^a^ Participants with miscarriage excluded.

Table 3 synthesises articles reporting on rupture rates with second‐trimester MToP using prostaglandins [8, 49, 50, 51, 52, 53, 54, 55, 56, 57, 58, 59, 60, 61, 62, 63, 64, 65, 66, 67, 68, 69, 70, 71, 72, 73, 74, 75, 76, 77, 78, 79, 80, 81, 82, 83, 84, 85, 86, 87, 88, 90, 91, 92, 93, 94, 95, 96, 97, 98, 99, 100, 101, 102, 103, 104, 105, 106, 107, 108, 109, 110, 111, 112, 113, 115, 116, 117]. Studies were excluded if ruptures occurred in the context of intravenous oxytocin use [66, 118, 119, 120, 121], included data from participants of unclear gestation (≤ 28 weeks vs. > 28 weeks), or did not specify a rupture rate [120, 122, 123, 124, 125, 126, 127, 128, 129, 130].

**TABLE 3:** Rupture rates by prostaglandin and number of CS in second‐trimester MToP.

Author/publication date	Number of participants	Method of abortion	Gestation	Number of participants with 1 prior CS^a^	Number of participants with 2(+) prior CS^a^	Number of participants with 3(+) prior CS^b^	Number of ruptures amongst those with 1 prior LSCS	Number of ruptures amongst those with ≥ 2 prior LSCS	Number of ruptures in those with prior classical CS	Rupture rate overall
Abou Elela [49] (2022)	50	50 μg PV misoprostol every 4 h	13–26 weeks	50			0			0 of 50
Aydin et al. [50] (2019)	85	50 μg PV misoprostol every 6 h until regular contractions	19.19 ± 2.63 weeks^c^							1 of 85
Bahar et al. [51] (2021)	128	Misoprostol 800 μg PV followed by 400 μg PO 3 hourly; max of 4 PO doses (IV oxytocin used after 3‐h interval if abortion not complete after misoprostol regimen)	13–26 weeks	99	15	14	1	0		1^d^ of 128
Basu et al. [52] (2009)	47	400 μg PV misoprostol every 8 h for up to 48 h; MVA following successful abortion prior to discharge	16 ± 2 weeks^c^							0 of 47
Berghella et al. [8] (2009)	17	100–800 μg PV misoprostol every 4–6 h	16–28 weeks	13	2		0	0	1 of 2	1 of 17
Bhattacharjee et al. [53] (2007)	80	200–400 μg PV/SL misoprostol every 4 h; max 24 h	13–26 weeks							0 of 80
Bhuvaneswari et al. [54] (2020)^e^	50	(1) Foley catheter + 200–400 μg PV misoprostol every 4 h; max 5 doses or	13–26 weeks	50			0			0 of 50
Bhuvaneswari et al. [54] (2020)^e^	50	(2) 200 mg mifepristone followed by 200–400 μg PV misoprostol every 4 h; max 5 doses	13–26 weeks	50			1			1 of 50
Brouns [55] (2010)	12	200 mg mifepristone followed by 200 μg or 400 μg PV misoprostol every 4 h	14–24 weeks							0 of 12
Cetin et al. [56] (2016)	77	(1) misoprostol 100 μg PV misoprostol every 4 h (2) misoprostol 200 μg SL every 3 h; max 5 doses	12–24 weeks	48	29		0	1		1 of 77
Chen [57] (2023)	204	200 mg PO mifepristone followed by various doses of PV/PO misoprostol ± cervical ripening balloon	14–27 weeks							1 of 204
Choudhary et al. [58] (2011)^f^	29	200 μg PV misoprostol every 4–6 h; max 24 h ± mifepristone	12–20 weeks							1 of 29
Daponte et al. [59] (2006)	85	400 μg PV misoprostol followed by 200–400 μg every 6 h; max 1600 μg	14–20 weeks	85			0			0 of 85
Daponte et al. [60] (2007)	21	400 μg PV misoprostol followed by 200 μg every 6 h; max 800 μg	1st and 2nd trimester^c^, ^g^		19	2		0		0 of 21
Daskalakis et al. [61] (2005)	108	400 μg PO misoprostol + 400 μg PV misoprostol followed by 400 μg PV misoprostol every 6 h; max 5 doses	17–24 weeks	96	11	1	0	0		0 of 108
Davey et al. [62] (1997)	22	600 mg mifepristone followed by 1 mg PV gemeprost every 3 h; max 5 doses	12–24 weeks							1 of 22
Dickinson et al. [63] (2005)	101	200 μg PV misoprostol every 6 h	14–28 weeks	78	19	4	0	0		0 of 101
Dickinson et al. [64] (2010)^e^	39	(1) 400 μg PV misoprostol every 6 h; max 48 h	14–28 weeks							0 of 39
Dickinson et al. [64] (2010)^e^	42	(2) 200 mg mifepristone followed by 800 μg PV misoprostol then 400 μg PO misoprostol every 3 h; max 5 doses	14–28 weeks							0 of 42
Dickinson et al. [65] (2023)^h^	304	200 mg PO mifepristone followed by 200–600 μg PV misoprostol every 3–4 h	14–28 weeks	241	62		3	1	0 of 1	4 of 304
Domrose et al. [66] (2012)^i^	100	1 mg gemeprost PV every 6 h	14–28 weeks	88	12		N/A^l^	N/A^l^		1 of 100
Elasy [67] (2022)	79	400 μg PV misoprostol followed by 200 μg misoprostol every 4 h	14–24 weeks							3 of 79
El‐Sayed [68] (2023)	28	400 μg PV misoprostol every 3–4 h	13–18 weeks	28			0			0 of 28
El Sharkwy et al. [69] (2019)	78	(1) 100 μg PV misoprostol 6 hourly (2) Intracervical Foley catheter with 30 mL Normal Saline + 100 μg PV misoprostol every 6 h	20–27 weeks		36	42		0		0 of 78
Ercan et al. [70] (2016)	144	(1) 200 μg PV misoprostol then 200 μg SL misoprostol every 4 h (2) 200 μg PV misoprostol with weighted intracervical Foley catheter 2 h later; if undelivered at 24 h 200 μg misoprostol every 4 h	14–24 weeks							0 of 144
Erturk et al. [71] (2022)	137	(1) 400 μg PV/SL misoprostol every 3–6 h (2) 400 μg PV/SL misoprostol followed by 200 μg every 3–6 h	14–24 weeks	79	58		0	1		1 of 137
Fawzy et al. [72] (2010)	31	200 μg PV misoprostol every 6 h; 400 μg PV misoprostol every 6 h beyond 24 h	13–26 weeks			31		1		1 of 31
Garofalo et al. [73] (2018)	141	SToP or MToP depending on patient preference and clinical expertise; MToP > 16 + 0 600 mg mifepristone followed by 1 mg gemeprost every 3 h	< 22 weeks^c^							6 of 141
Gomez et al. [74] (2010)	28	200 mg PO mifepristone followed by 800 μg PV misoprostol then 400 μg misoprostol every 3 h	12–23 weeks							0 of 28
Gulec et al. [75] (2013)	86	200 μg PV misoprostol 4 hourly; max 1200 μg	14–26 weeks	60	26		0	3		3 of 86
Henkel [76] (2020)	5	200 mg PO mifepristone followed by 400 μg buccal misoprostol every 3 h	15–27 weeks							0 of 5
Herabutya et al. [77] (2003)	56	600–800 μg PV misoprostol every 6–12 h	14–26 weeks	45	8	3	0	0		0 of 56
Hou et al. [78] (2010)	5	200 mg PO mifepristone followed by 400 μg PO misoprostol every 6 h	13–16 weeks							0 of 5
Jacques et al. [79] (2020)	13^j^	200 mg mifepristone followed by 400 μg PV or SL misoprostol	14–24 weeks							0 of 13
Jamali et al. [80] (2020)	431	100–400 μg misoprostol PV every 4–6 h	14–24 weeks	218	213		3	7		10 of 431
Kapp et al. [81] (2007)	3	200 mg PO mifepristone followed by 400 μg buccal misoprostol then 200 μg buccal misoprostol every 6 h	18–23 weeks							0 of 3
Kiley et al. [82] (2022)	3	Feticide followed by 200 μg misoprostol every 4 h	23–26 weeks	3			0			0 of 3
Koh et al. [83] (2018)	339	(1) Gemeprost PV 1 mg or (2) misoprostol PO 400 μg every 4 h; max 5 doses	14–23 weeks							7 of 339^k^
Latta et al. [84] (2023)^e^	77	800 μg misoprostol then 400 μg PV/SL every 3 h	14–24 weeks	55	22		0	2	0 of 11	2 of 77
Latta et al. [84] (2023)	32	PO mifepristone followed by 800 μg misoprostol then 400 μg PV/SL every 3 h	14–24 weeks	22	10		0	0		0 of 32
Liaquat et al. [85] (2006)	5	50 μg PV misoprostol every 4 h; max 4 doses; followed by syntocinon if abortion not complete	14–26 weeks							0 of 5
Marinoni et al. [86] (2007)	62	1 mg PV gemeprost every 3 h; max 5 doses	13–23 weeks	52	8	2	N/A^l^	N/A^l^		0 of 62
Masse et al. [87] (2020)	51	200 mg mifepristone^m^ then 200–400 μg misoprostol; various routes and frequencies	14–24 weeks							1 of 51^n^
Mazouni et al. [88] (2006)	50	15–34 weeks: 600 mg mifepristone followed by 200–400 μg PV misoprostol every 3 h; or > 34 weeks mifepristone followed by Prostin E2 gel	> 15 weeks^c^							2 of 50
Meaidi [89] (2020)	236	200 mg mifepristone followed by 400 μg PV misoprostol every 3 h	13–23 weeks							2 of 236
Mobusher [90] (2013)	100	400 μg PV misoprostol every 6 h; max 5 doses	14–24 weeks	95	4	1	0	0		0 of 100
Morra et al. [91] (2019)	340	1 mg PV gemeprost every 3 h; max 5 doses ± 200 mg PO mifepristone prior	13–24 weeks							9 of 340
Munir et al. [92] (2014)^o^	50	5× doses 200 μg PO misoprostol every 6 h	13–26 weeks							0 of 50
Naguib et al. [93] (2010)	50	200 μg PV misoprostol 4 hourly	16–26 weeks	50			0			0 of 50
Obata‐Yasuoka et al. [94] (2009)	26	Laminaria 4 hourly for 2 days; on day 3 removal of laminaria + 1 mg PV gemeprost every 3 h; max 4 doses	12–21 weeks	19	7		N/A^l^	N/A^l^		0 of 26
Peng et al. [95] (2015)^p^	32	200 mg PO mifepristone followed by 600 μg misoprostol then 200 μg PV misoprostol every 3 h; max 1800 μg daily	13–16 weeks							1 of 32
Petca [96] (2019)	7	200 mg mifepristone followed by 400 μg PV misoprostol then 50 μg SL and 50 μg PV every 6 h	16–24 weeks							0 of 7
Pongsatha et al. [97] (2011)	64	400–800 μg PV/PO misoprostol every 3–12 h	20.9 ± 3.9^c^							0 of 64
Pongsatha at al [98] (2024)	80	400 μg PV misoprostol every 6 h	14–28 weeks							1 of 80
Pourhouseini et al. [99](2023)	104	(1) letrozole 2.5 mg every 6 h total of 6 doses prior to misoprostol (2) various doses of PV misoprostol	< 20 weeks^c^							0 of 104
Reehan [100] (2024)	100	100–400 μg PV misoprostol every 3 h	13–26 weeks		60	40		2		2 of 100
Reischer [101] (2023)	139	600 mg PO mifepristone followed by 400 μg PO misoprostol every 3 h; feticide if > 22 weeks	14–24 weeks							0 of 139
Scioscia et al. [102] (2005)^q^	63	1 mg PV gemeprost every 3 h; max 3 doses per day	13–23 weeks	55	5	1	N/A^l^	N/A^l^		0 of 63
Shammas et al. [103] (2006)	63	400 μg PV misoprostol followed by 200 μg PV misoprostol every 6 h	15–28 weeks	38	15	10	0	0		0 of 63
Shantikumar et al. [104] (2021)	20	200 mg mifepristone day 1 and repeat dose day 2, followed by 200–400 μg PV misoprostol 6 hourly	13–20 weeks	20			0			0 of 20
Sharma et al. [105] (2020)	6	200 mg PO mifepristone followed by 400 μg SL every 3 h	13–28 weeks	6			0			0 of 6
Shay et al. [106] (2022)^e^	15	200 mg mifepristone followed by 400–600 μg PV misoprostol every 3–6 h	14–28 weeks							0 of 15
Shay et al. [106] (2022)^e^	35	400–600 μg PV misoprostol every 3–6 h	14–28 weeks							0 of 35
Stewart et al. [107] (2022)	72	200 mg mifepristone followed 25–200 μg misoprostol PV or PO; max 4 doses	≥ 20 weeks (average 23 weeks; IQR 22–26) ^c^	50	22	3	2	0	0 of 1	2 of 75
Tarim et al. [108] (2005)	12	200 μg PO misoprostol hourly; max 6 doses	2nd trimester^c^	12			0			0 of 12
Torriente et al. [109] (2017)	268	800 μg PV misoprostol followed by 200 μg every 2 h; max 1400 μg	13–20 weeks	231	37		0	0		0 of 268
Turgut et al. [110] (2013)	56	50–600 μg PV misoprostol every 4–6 h	13–24 weeks							3 of 56
van Bogaert [111] (2007)^r^	18	400 μg SL misoprostol + 800 μg PO misoprostol	< 20 weeks^c^							0 of 18
Velipasaoglu et al. [112] (2018)	104	200 μg PV misoprostol every 4 h; if cervix not favourable at 24 h then Foley catheter inserted	14–22 weeks	88	16		0	0		0 of 104
Vlad et al. [113] (2022)	9	200 mg PO mifepristone followed by 400 μg PV misoprostol every 4 h	13–24 weeks							0 of 9
Totals	5604			2124	708	154	10 of 1910 (0.52%)^a^	18 of 835 (2.16%)^a^	1 of 15 (6.66%)	69 of 5604 (1.23%)
[95% CI]							[0.28%–0.97%]	[1.36%–3.40%]	[1.00%–44.28%]	[1.00%–1.56%]
Misoprostol alone	3198									31 of 3198 (0.97%)
[95% CI]										[0.68–1.38]
Mifepristone + misoprostol	1313									14 of 1314 (1.07%)
[95% CI]										[0.63–1.79]
Gemeprost	1093									24 of 1093 (2.20%)
[95% CI]										[1.48–3.26]

Abbreviations: CI, confidence interval; CS, caesarean section; IV, intravenous; LSCS, lower segment caesarean section; MToP, medical termination of pregnancy; MVA, manual vacuum aspiration; PO, per oral; PV, per vagina; SL, sublingual; SToP, surgical termination of pregnancy.

^a^ Data on the number of prior CS were not provided in many of the articles; where these data were provided, it has been entered into this table. Due to missing data, total ruptures for those with 1 prior and 2 prior CS do not equal the total rupture rates provided, which include the majority of participants for whom the number of prior CS was not reported.

^b^ There was insufficient available data on ruptures amongst those documented to have had 3+ prior CS; however, the number of included participants [114] is included in the table and highlights the lack of data in this subgroup.

^c^ Exact gestational range not provided.

^d^ Rupture was in the misoprostol group.

^e^ Data from study separated into two different treatment groups for analysis.

^f^ Excluded women managed with oxytocin or planned hysterotomy [58].

^g^ Unable to exclude first trimester cases.

^h^ Dickinson et al. (2014) not included in this analysis as it captures the same cases of rupture.

^i^ Excluded women in the study who were in the third trimester.

^j^ Numbers not clear from data provided in article; however, data obtained from Henkel et al. 2023 systematic review paper.

^k^ All cases of rupture received gemeprost.

^l^ Only misoprostol MToP is included in the analysis of rupture by number of previous CS; studies using gemeprost were excluded.

^m^ Only 2.1% of this cohort used mifepristone.

^n^ This patient had not used mifepristone prior to misoprostol.

^o^ Excluded women who underwent instillation abortion and oxytocin (100 additional women with no ruptures); unable to separate out numbers on women with 2 and 3+ prior CS from the group that received misoprostol, however this study included in total: 66 with 1 prior CS, 49 with 2 prior CS and 35 with 3+ prior CS.

^p^ Only 32 women received mife/miso, and these were included in the analysis; others received ethacridine lactate and were excluded from the analysis.

^q^ Includes 2 women with previous transmural myomectomy.

^r^ Only 18 women in the second trimester.

Sixty‐six studies (5604 women) with previous CS undergoing second‐trimester prostaglandin MToP were included in the analysis: 69 ruptures occurred (1.23%, 95% CI 1.00%–1.56%), 45 of 4512 with misoprostol use (1.00%, CI 0.75%–1.33%) and 24 of 1093 with gemeprost (2.20%, CI 1.48%–3.26%; *p* = 0.001). Of the 69 ruptures, the mean gestation was 21 weeks (SD 3.8), and 53 (76.8%) were managed with closure of the defect by laparoscopy or laparotomy without hysterectomy.

Rupture rates were also calculated by number of previous CS where data was available (Table 3), demonstrating a rupture rate of 10 of 1910 (0.52%, CI 0.28%–0.97%) after 1 previous CS, and 18 of 835 (2.16%, CI 1.36%–3.40%) after ≥ 2 previous CS (*p* < 0.001).

There was no difference in rupture rates between misoprostol alone (1.0%) compared with the use of mifepristone and misoprostol (1.1%; *p* = 0.77). However, numerous studies reported a reduction in abortion time with the addition of mifepristone 24–48 h prior to misoprostol [54, 81, 96, 106, 115]. There were significant variations in misoprostol dosage, timing and route of administration. Where dosage data were able to be extracted and categorised into low‐dose only (≤ 200 μg) and > 200 μg increments, studies that used low‐dose increments only [49, 50, 56, 58, 63, 69, 70, 75, 82, 85, 92, 93, 107, 108, 112, 115] did not have significantly lower rupture rates than those using > 200 μg [51, 52, 59, 60, 61, 64, 67, 68, 71, 74, 76, 77, 78, 79, 81, 84, 85, 90, 95, 97, 98, 101, 103, 105, 106, 109, 111, 113, 115, 117, 131] (8/843 (0.95%) and 11/1963 (0.56%) respectively, *p* = 0.08).

### 3.2 Efficacy of MToP After Previous Caesarean

Thirty‐eight studies reported on the efficacy of MToP in 12,177 women with and 95,122 without previous CS [42, 48, 50, 51, 52, 58, 61, 63, 65, 72, 75, 77, 80, 84, 85, 86, 88, 90, 94, 98, 102, 103, 109, 110, 112, 117, 118, 120, 125, 127, 131, 132, 133, 134, 135, 136, 137]. Across both trimesters, the risk of failed MToP/need for surgical intervention was higher in women with previous CS (OR 1.48, CI 1.29–1.70) (Figure S1). This was still significant when analysed separately for the first (OR 1.88, CI 1.14–2.52) or second trimester (OR 1.32, CI 1.11–1.56).

Two studies examined the ability of ultrasound of CS scar thickness to predict the outcome of MToP. A retrospective review of 183 women with previous CS undergoing first trimester MToP found that a CS scar defect on transvaginal ultrasound, where residual myometrial thickness was < 30% of the adjacent myometrial thickness, had an increased chance of needing surgical intervention (OR 3.32, CI 1.64–6.75) with an overall risk of 57.1% if myometrial thickness ratio < 30% [138]. A small study including 66 women demonstrated that a lower uterine segment thickness < 3 mm was associated with uterine rupture (OR 94, CI 4.2–2106) [95].

### 3.3 Safety of SToP After Previous Caesarean

There were two case reports of perforation through CS scar during SToP [139, 140].

Eight studies, including 542 women with and 10,979 women without previous CS, reported on the safety and efficacy of cervical priming prior to SToP [141, 142, 143, 144, 145, 146, 147, 148]. Laminaria and/or misoprostol in doses varying from 100 to 800 μg were used with no cases of uterine rupture.

Eleven studies including 2760 women with previous CS reported on adverse events during SToP [141, 146, 149, 150, 151, 152, 153, 154, 155, 156, 157]. Adverse outcomes were more common across both trimesters amongst women with previous CS (OR 2.43, CI 1.56–3.78). Only one retrospective cohort study reported on outcomes of first‐trimester SToP, demonstrating an increased risk of complications associated with previous CS (OR 1.9, CI 1.1–3.4) [150]; all other studies included second‐trimester procedures or a combination of first‐ and second‐trimester cases.

### 3.4 Termination of Pregnancy After Previous Classical Caesarean

Fifteen women with previous classical CS were identified within the original research papers, among whom only one experienced rupture (6.66%, CI 1.00%–44.28%). See Table 3.

### 3.5 Termination of Pregnancy in the Context of Abnormal Placentation

Eleven case reports described undiagnosed CSP or PAS encountered during abortion (6–18 weeks gestation) [36, 114, 158, 159, 160, 161, 162, 163, 164]. One report described an undiagnosed arteriovenous malformation (AVM) at the site of a CS scar in a woman with four previous CS; this was diagnosed with angiography after large haemorrhage during a 12‐week SToP [165]. In all cases, ultrasound had been used, failing to recognise abnormal implantation of trophoblastic tissue. In 8 of 11 reports of undiagnosed CSP/PAS, hysterectomy was required to control haemorrhage; 5 of 11 reported massive blood loss.

Eight case reports of second‐trimester abortion with known PAS were identified [32, 166, 167, 168, 169, 170, 171], describing several management techniques. In five cases, magnetic resonance imaging (MRI) confirmed the diagnosis. Surgical management included gravid hysterectomy, D&E or planned hysterotomy. Adjunct methods to improve safety included methotrexate and/or uterine artery embolisation (UAE) [166, 168]. Three cases describe medical management alone using either feticide and methotrexate, mifepristone/misoprostol or gemeprost [32, 166, 170]. All three cases required surgical intervention and described significant complications.

Nine original studies (160 women) described abortion in those with previous CS and either PAS or placenta praevia [172, 173, 174, 175, 176, 177, 178, 179, 180]. One series of seven women with undiagnosed PAS at D&E requiring hysterectomy to control bleeding [176] affirms case reports demonstrating a high risk of haemorrhage and emergency hysterectomy. In another study, four cases of undiagnosed PAS during first‐trimester SToP experiencing haemorrhage were successfully managed with uterine artery embolisation (UAE) with uterine preservation [174]. There was successful use of UAE prior to MToP or hysterotomy in 12 patients undergoing mid‐trimester abortion with PAS, showing a reduction in mean blood loss from 1533 to 383 mL [178]. Seven cases of mid‐trimester hysterotomy and internal iliac ligation with accreta are reported, with prophylactic UAE; all experienced massive haemorrhage and almost half required emergency hysterectomy [172]. The largest study identified on this topic is from China and describes the management of 51 people with PAS undergoing mid‐trimester MToP; 31 had UAE followed by MToP and 20 had UAE followed by planned hysterotomy [173]. Two thirds having MToP required curettage for abnormally adherent placental tissue; however, only 7.8% required hysterectomy and there was no difference between MToP and hysterotomy in terms of blood loss, transfusion, hospital stay duration or need for hysterectomy [173]. Placenta praevia without PAS was significantly associated with the need for emergency UAE and intensive care admission in one study with 34 cases for abortion in the setting of placenta praevia [180]. Adjunct measures described in these studies to reduce blood loss include internal iliac ligation, intrauterine balloon tamponade and adjunct methotrexate.

### 3.6 Critical Appraisal

Results of the risk of bias and quality appraisal of original studies are presented in Appendix S2.

## 4 Discussion

### 4.1 Main Findings

This review summarises the large and rapidly growing body of evidence regarding the management of induced abortion in people with previous CS. Uterine rupture is rare during the first trimester, with only three case reports identified [24, 33, 40], and no ruptures in observational studies [42, 43, 44, 45, 46, 47, 48]. Perdue et al. recently reviewed 61 cases of first‐trimester rupture reported in the literature, of which 30% required hysterectomy; however, none were in the setting of induced abortion [181]. The findings of this scoping review support the safety of first‐trimester MToP outside hospital settings for women with previous CS.

In contrast, CSP is increasingly reported [14], and has the potential for significant morbidity if undetected prior to abortion. In 1995, Rashbaum reported the incidence of undiagnosed accreta encountered at second trimester SToP to be 0.04% [176]; however, the current incidence is likely considerably higher, given the incidence of PAS rose fourfold 1994–2002 [182]. Most case reports of undiagnosed CSP occurred during the first trimester; associated morbidity was high, with a significant risk of haemorrhage and a need for hysterectomy. Ultrasound is not always reliable for identifying abnormal placentation in early pregnancy, when reported sensitivity and specificity for PAS are 41% and 88%, respectively [183]; thus, the optimal imaging modality for ruling out CSP/PAS prior to abortion remains unclear. MRI has not reliably been shown to have improved sensitivity or specificity compared to ultrasound in diagnosing PAS but can be a useful adjunct to ultrasound, the latter still considered first line [184]. Pre‐abortion ultrasound, including assessment for CSP/PAS, should be recommended for all women undergoing pregnancy termination on the background of a prior caesarean section. Detection rates vary depending on gestation and operator experience and are higher when performed by experts. Due to the relatively uncommon nature of the condition and the absence of specific sonographer credentialling in PAS [184], it would seem reasonable for expert/tertiary ultrasound to be sought prior to abortion for women at significantly increased risk, such as those with ≥ 3 previous CS, or in cases where ultrasound demonstrates a gestational sac sitting low or near the CS scar [185]. Furthermore, failed MToP or ongoing bleeding after SToP in those with prior CS should alert the clinician to the possibility of CSP [114, 159, 163].

This review contains the largest cumulative meta‐analysis of uterine rupture rates during prostaglandin MToP to date. In 2009, two systematic reviews of misoprostol MToP reported similarly low rupture rates of 0.28% and 0.43% (among 722 and 507 women with previous CS) [6, 8]. At this time, available studies only included a total of 46 women with two previous CS, making it difficult to draw conclusions about the risk of rupture in this group [8]. A recent systematic review by Henkel et al. reported a rupture rate of 1.1% among 876 women undergoing second‐trimester MToP with mifepristone and misoprostol [9]. Our review similarly shows a higher rupture rate (1.0%) with misoprostol regimens in the second trimester than that published in earlier meta‐analyses, but our review suggests that mifepristone‐misoprostol compared to misoprostol alone shortens abortion time without increasing the risk of rupture. This updated rupture rate is closer to term induced vaginal birth after one previous CS [7]; however, in the context of an abortion, fetal hypoxia is not of concern. There remains a risk of significant maternal haemorrhage, and uterine rupture should be treated as an emergency; however, it is reassuring that a majority (76%) of ruptures were managed without hysterectomy, and that laparoscopic techniques for repair are being reported.

Owing to the rise in CS rate and the large numbers of studies published on this topic since previous reviews, our review included significantly more women with ≥ 2 previous CS (*n* = 835) than previously published; this reveals that a history of ≥ 2 previous CS is associated with increased risk of rupture compared to women with a single prior CS. It is likely that women with ≥ 3 CS are at increasingly higher risk of rupture, although available evidence remains insufficient for accurate analysis. This requires further research and raises the question of whether surgical termination is safer than medical termination in women with multiple previous CS.

There was significant heterogeneity in relation to dose, intervals and mode of administration of misoprostol for second‐trimester MToP. Most studies used vaginal or sublingual administration, which is associated with fewer side effects and better absorption than oral [186, 187], and Dickinson found that 400 μg shortened abortion time compared with 200 μg doses [115]. Sublingual or buccal administration has similar pharmacokinetics to vaginal and was used in some studies [188]. Further research is required to determine whether there is benefit to reduction in dose of misoprostol in women with prior CS, as recommended by some guidelines [12].

Gemeprost was associated with a higher rupture rate than misoprostol (2.20% vs. 1.00%, *p* < 0.001). Furthermore, Le Roux showed mifepristone‐misoprostol to be significantly more effective at achieving complete abortion compared to gemeprost (94% vs. 68%, *p* = 0.02) [126]. Misoprostol is the most commonly used prostaglandin for abortion and should be the preferred choice.

To our knowledge, this is the first data synthesis showing previous CS was associated with moderately increased risk of retained products of conception and/or need for surgical intervention. Mifepristone shortens abortion time and is routinely used in many countries prior to misoprostol for MToP [128]; it appears safe in both first and second trimesters, decreases abortion time and may reduce the incidence of incomplete abortion [64].

The absence of rupture amongst 542 women with mechanical and/or prostaglandin ripening prior to D&E is reassuring. Although uncommon, rupture in this setting is possible, as highlighted by the four individual case reports of uterine rupture from cervical ripening prior to D&E [23, 34, 38]. The largest study in the first trimester showed a significant reduction in the need for mechanical dilatation with the use of low dose misoprostol [144]. Hern published a non‐blinded controlled clinical trial of feticide and laminaria with and without additional misoprostol prior to late D&E, showing that adding misoprostol reduced procedure length and blood loss; however, previous CS was a risk factor for haemorrhage (*p* < 0.0001) [143]. A smaller retrospective study found no difference in efficacy between overnight osmotic dilators and misoprostol 1‐h prior to D&E [147]. Importantly, Ben‐Ami and associates found that previous CS was a significant risk factor for inadequate dilation prior to D&E [189]. Given that difficult or inadequate dilatation is a risk factor for complications such as perforation, further clarification on optimal ripening pre‐procedures for women with previous CS is warranted.

Classical CS is known to increase rupture risk with subsequent labour compared to LSCS [190], and is considered a contraindication to a trial of labour at term [191]. Seto's case report is accompanied by a comprehensive literature review on MToP after classical CS, reporting only 16 cases ever published, two of which were complicated by rupture [192]. Several of these reports were regarding spontaneous mid‐trimester labour, fetal death, or instillation abortion and hence are not included in our review [193, 194, 195, 196, 197]. Our review includes 15 women with previous classical CS, with one uterine rupture. It remains unclear whether surgical abortion is a safer option for women with prior classical CS, and evidence is likely to remain predominantly based on case reports and expert consensus given the infrequency of classical CS.

There is a paucity of literature on the management of abortion for women with PAS. The available evidence, largely from case series, describes various techniques including medical and surgical (hysterotomy), with adjunctive UAE to reduce blood loss and the need for hysterectomy. There remain theoretical concerns regarding the reduction in uterine vascularity and the risk of growth restriction in pregnancies following UAE; however, subsequent successful pregnancies at term have been reported [198]. Additionally, important is the risk of recurrence of PAS in subsequent pregnancies [14], counselling is required and for those who do not desire future fertility, gravid hysterectomy with or without prophylactic UAE could be considered. Further evidence is required on this topic, and management should be individualised.

This review is limited by the exclusion of non‐English articles; however, it was broadened by having no date limitations. Regardless, it captures data from across the globe (31 countries). Due to the heterogeneity of methodology and aims of available research, some data were unavailable for extraction. Despite excluding papers only including women with miscarriage and IUFD, some included papers contained both abortions and pregnancy loss cases, and abortion‐only data were unable to be extracted separately.

This scoping review offers insights into the increasingly important topic of abortion complexities after previous caesareans and provides avenues for further research. Prior caesarean delivery increases the risk of adverse maternal outcomes in women having abortion. In particular, second‐trimester abortion care should be provided by experienced health care providers with the knowledge and available infrastructure to provide high‐level care if difficulties are encountered.

## Conflicts of Interest

The authors declare no conflicts of interest.

## Supporting information


Appendix S1

Appendix S2

Figure S1

Table S1

Table S2

Table S3

Table S4

Table S5

## References

[1] A. P. Betran , J. Ye , A.‐B. Moller , J. P. Souza , and J. Zhang , “Trends and Projections of Caesarean Section Rates: Global and Regional Estimates,” BMJ Global Health 6, no. 6 (2021): e005671. 10.1136/bmjgh-2021-005671 PMC8208001 34130991

[2] Australian Institute of Health and Welfare , “Australia's Mothers and Babies,” 2022.

[3] Health AIo, Welfare , National Core Maternity Indicators (AIHW, 2023).

[4] A. Chan and L. C. Sage , “Estimating Australia's Abortion Rates 1985‐2003,” Medical Journal of Australia 182, no. 9 (2005): 447–452. 15865587 10.5694/j.1326-5377.2005.tb06783.x

[5] T. Eshkoli , A. Y. Weintraub , R. Sergienko , and E. Sheiner , “Placenta Accreta: Risk Factors, Perinatal Outcomes, and Consequences for Subsequent Births,” Obstetric Anesthesia Digest 34, no. 1 (2014): 19–20. 10.1016/j.ajog.2012.12.037 23313722

[6] V. Goyal , “Uterine Rupture in Second‐Trimester Misoprostol‐Induced Abortion After Cesarean Delivery: A Systematic Review,” Obstetrics and Gynecology 113, no. 5 (2009): 1117–1123. 19384128 10.1097/AOG.0b013e31819dbfe2

[7] G. A. Dekker , A. Chan , C. G. Luke , et al., “Risk of Uterine Rupture in Australian Women Attempting Vaginal Birth After One Prior Caesarean Section: A Retrospective Population‐Based Cohort Study,” BJOG: An International Journal of Obstetrics and Gynaecology 117, no. 11 (2010): 1358–1365, 10.1111/j.1471-0528.2010.02688.x. 20716251

[8] V. Berghella , J. Airoldi , A. O'Neill , K. Einhorn , and M. Hoffman , “Misoprostol for Second Trimester Pregnancy Termination in Women With Prior Caesarean: A Systematic Review,” BJOG: An International Journal of Obstetrics and Gynaecology 116, no. 9 (2009): 1151–1157, 10.1111/j.1471-0528.2009.02190.x. 19438490

[9] A. Henkel , H. E. Miller , J. Zhang , D. J. Lyell , and K. A. Shaw , “Prior Cesarean Birth and Risk of Uterine Rupture in Second‐Trimester Medication Abortions Using Mifepristone and Misoprostol: A Systematic Review and Meta‐Analysis,” Obstetrics and Gynecology 142, no. 6 (2023): 1357–1364. 37884011 10.1097/AOG.0000000000005259

[10] National Institute for Health and Care Excellence , “Abortion Care [Nice Guideline No. 140],” 2019.

[11] World Health Organization , Abortion Care Guideline (World Health Organization, 2022).

[12] Queensland Clinical Guidelines , Termination of Pregnancy, Guideline No. MN19.21‐V6‐R24 (Queensland Health, 2020).

[13] M. Morlando , L. Sarno , R. Napolitano , et al., “Placenta Accreta: Incidence and Risk Factors in an Area With a Particularly High Rate of Cesarean Section,” Acta Obstetricia et Gynecologica Scandinavica 92, no. 4 (2013): 457–460. 23347183 10.1111/aogs.12080

[14] N. Drever , J. Bertolone , M. Shawki , and S. Janssens , “Caesarean Scar Ectopic Pregnancy: Experience From an Australian Tertiary Centre,” Australian and New Zealand Journal of Obstetrics and Gynaecology 60, no. 3 (2020): 330–335. 31944267 10.1111/ajo.13119

[15] C. Pawliuk , H. L. Brown , K. Widger , et al., “Optimising the Process for Conducting Scoping Reviews,” BMJ Evidence‐Based Medicine 26, no. 6 (2020): 312, 10.1136/bmjebm-2020-111452. 33087454

[16] E. Aromataris and Z. Munn , JBI Manual for Evidence Synthesis (JBI, 2020).

[17] A. C. Tricco , E. Lillie , W. Zarin , et al., “PRISMA Extension for Scoping Reviews (PRISMA‐ScR): Checklist and Explanation,” Annals of Internal Medicine 169, no. 7 (2018): 467–473, 10.7326/m18-0850. 30178033

[18] J. A. C. Sterne , J. Savović , M. J. Page , et al., “RoB 2: A Revised Tool for Assessing Risk of Bias in Randomised Trials,” BMJ 366 (2019): l4898‐l. 31462531 10.1136/bmj.l4898

[19] J. A. C. Sterne , M. A. Hernán , B. C. Reeves , et al., “ROBINS‐I: A Tool for Assessing Risk of Bias in Non‐Randomised Studies of Interventions,” BMJ 355 (2016): i4919. 27733354 10.1136/bmj.i4919 PMC5062054

[20] ROBINS‐E Development Group , J. M. R. Higgins , A. Rooney , et al., “Risk of Bias in Non‐Randomized Studies—Of Exposure (ROBINS‐E) Launch Version, 1 June 2022,” https://www.riskofbias.info/welcome/robins‐e‐tool.

[21] A. Agresti and B. A. Coull , “Approximate Is Better Than “Exact” for Interval Estimation of Binomial Proportions,” American Statistician 52, no. 2 (1998): 119–126.

[22] M. J. Page , J. E. McKenzie , P. M. Bossuyt , et al., “The PRISMA 2020 Statement: An Updated Guideline for Reporting Systematic Reviews,” BMJ 372 (2021): n71, 10.1136/bmj.n71. 33782057 PMC8005924

[23] L. Berghahn , D. Christensen , and S. Droste , “Uterine Rupture During Second‐Trimester Abortion Associated With Misoprostol,” Obstetrics and Gynecology 98, no. 5 (2001): 976–977, 10.1016/s0029-7844(01)01546-0. 11704229

[24] O. Bika , D. Huned , S. Jha , and K. Selby , “Uterine Rupture Following Termination of Pregnancy in a Scarred Uterus,” Journal of Obstetrics and Gynaecology 34, no. 2 (2014): 198–199. 24456452 10.3109/01443615.2013.841132

[25] G. Caruso , V. Paladini , V. D'Ambrosio , et al., “Combined Vesicouterine Rupture During Second‐Trimester Medical Abortion for Fetal Abnormality After Prior Cesarean Delivery: A Case Report. Case Reports,” Women's Health 32 (2021): e00364. 10.1016/j.crwh.2021.e00364 PMC8570940 34765461

[26] M. Chen , J. C. Shih , W. T. Chiu , and F. J. Hsieh , “Separation of Cesarean Scar During Second‐Trimester Intravaginal Misoprostol Abortion,” Obstetrics and Gynecology 94, no. 5 SUPPL. 1 (1999): 840, 10.1016/s0029-7844(99)00335-x. 10546751

[27] M. Ciebiera , K. Zaręba , and G. Jakiel , “Laparoscopic Management of Uterine Cesarean Scar Dehiscence During Mid‐Trimester Misoprostol‐Induced Termination of Pregnancy,” Taiwanese Journal of Obstetrics & Gynecology 57, no. 4 (2018): 611–612. 30122590 10.1016/j.tjog.2018.04.031

[28] G. Daskalakis , N. Papantoniou , S. Mesogitis , J. Papageorgiou , and A. Antsaklis , “Sonographic Findings and Surgical Management of a Uterine Rupture Associated With the Use of Misoprostol During Second‐Trimester Abortion,” Journal of Ultrasound in Medicine 24, no. 11 (2005): 1565–1568. 16239663 10.7863/jum.2005.24.11.1565

[29] A. El‐Matary , R. Navaratnarajah , and D. L. Economides , “Ultrasound Diagnosis of Uterine Dehiscence Following Mifepristone/Misoprostol Regime in Early Second Trimester Termination,” Journal of Obstetrics and Gynaecology 26, no. 6 (2006): 578–580. 17000517 10.1080/01443610600830912

[30] F. Golshahi , F. Yarandi , S. Ramhormozian , and E. Shirali , “Lessons Learnt From Cases of Misoprostol‐Based Pregnancy Termination Followed by Uterine Rupture: Report of 3 Cases,” Journal of Obstetrics, Gynecology & Cancer Research 7, no. 2 (2022): 121–125.

[31] R. Gosakan , V. Ghule , H. H. Gergis , and E. Emovon , “Uterine Rupture Following a Second Trimester Medical Termination of Pregnancy in a Woman With a Previous Caesarean Section,” Journal of Obstetrics and Gynaecology 26, no. 8 (2006): 827–828. 17130055 10.1080/01443610600994783

[32] Q. Jiang , L. Yang , C. Ashley , E. E. Medlin , D. M. Kushner , and Y. Zheng , “Uterine Rupture Disguised by Urinary Retention Following a Second Trimester Induced Abortion: A Case Report,” BMC Women's Health 15, no. 1 (2015): 1. 25608736 10.1186/s12905-014-0159-9 PMC4310148

[33] E. Jwarah and J. O. Greenhalf , “Rupture of the Uterus After 800 Micrograms Misoprostol Given Vaginally for Termination of Pregnancy,” BJOG: An International Journal of Obstetrics and Gynaecology 107, no. 6 (2000): 807. 10847241 10.1111/j.1471-0528.2000.tb13346.x

[34] E. S. Lichtenberg and M. C. Frederiksen , “Cesarean Scar Dehiscence as a Cause of Hemorrhage After Second‐Trimester Abortion by Dilation and Evacuation,” Contraception 70, no. 1 (2004): 61–64. 15208054 10.1016/j.contraception.2004.02.013

[35] U. Nayki , C. E. Taner , T. Mizrak , C. Nayki , and G. Derin , “Uterine Rupture During Second Trimester Abortion With Misoprostol,” Fetal Diagnosis and Therapy 20, no. 5 (2005): 469–471. 16113576 10.1159/000087115

[36] R. Poudel , G. Dangal , A. Karki , et al., “Uterine Rupture During Medical Induction for Second Trimester Abortion,” Journal of Nepal Health Research Council 18, no. 2 (2020): 330–331. 32969405 10.33314/jnhrc.v18i2.2461

[37] U. Rajesh , S. Vyjayanthi , and N. Piskorowskyj , “Silent Uterine Rupture Following Second Trimester Medical Termination of Pregnancy in a Woman With an Artificial Urinary Sphincter and Three Previous Caesarean Sections,” Journal of Obstetrics and Gynaecology 22, no. 6 (2002): 687. 12554269 10.1080/014436102762062367

[38] M. L. Stitely , S. Craw , E. Africano , and R. Reid , “Uterine Scar Dehiscence Associated With Misoprostol Cervical Priming for Surgical Abortion: A Case Report,” Journal of Reproductive Medicine 60, no. 9–10 (2015): 445–448. 26592074

[39] E. Zohav , A. Alasbah , O. Segal , et al., “EP25.14: Ultrasound Finding of Rare Case of Early Second Trimester Uterine Rupture Following Misoprostol Administration in Patient With Previous One Caesarean Section,” Ultrasound in Obstetrics & Gynecology 48, no. S1 (2016): 375, 10.1002/uog.17145.

[40] C. Faraj , F. Chait , Y. Elharras , N. Allali , S. El Haddad , and L. Chat , “A Rare Case of Uterine Rupture in the First Trimester of Pregnancy: Case Report and Review of Literature,” Radiology Case Reports 19, no. 6 (2024): 2202–2205. 38515767 10.1016/j.radcr.2024.02.055 PMC10955100

[41] D. Limbachiya , R. Tiwari , and R. Kumari , “Laparoscopic Management of Second Trimester Vesico Uterine Rupture,” Journal of Obstetrics and Gynaecology of India 73, no. Suppl 2 (2023): 261–263. 38143990 10.1007/s13224-023-01795-3 PMC10746591

[42] L. W. Chien , W. M. Liu , C. R. Tzeng , H. K. Au , L.‐W. Chien , and W.‐M. Liu , “Effect of Previous Live Birth and Prior Route of Delivery on the Outcome of Early Medical Abortion,” Obstetrics & Gynecology 113, no. 3 (2009): 669–674, 10.1097/AOG.0b013e31819638e6. 19300333

[43] P. Gao and P. Wang , “Clinical Observation on Termination of Early Pregnancy of 213 Cases After Caesarian Section With Repeated Use of Mifepristone and Misoprostol,” Reproduction and Contraception 10, no. 4 (1999): 227–233. 12349659

[44] R. Gautam and V. Agrawal , “Early Medical Termination Pregnancy With Methotrexate and Misoprostol in Lower Segment Cesarean Section Cases,” Journal of Obstetrics and Gynaecology Research 29, no. 4 (2003): 251–256. 12959148 10.1046/j.1341-8076.2003.00108.x

[45] G. Wang , D. Li , F. Manconi , B. Dong , Y. Zhang , and B. Sun , “Timing and Indication for Curettage After Medical Abortion in Early Pregnant Women With Prior Uterine Incision,” Contraception 81, no. 1 (2010): 62–66. 20004275 10.1016/j.contraception.2009.09.013

[46] J. Xu , H. Chen , T. Ma , and X. Wu , “Termination of Early Pregnancy in the Scarred Uterus With Mifepristone and Misoprostol,” International Journal of Gynecology & Obstetrics 72, no. 3 (2001): 245–251. 11226445 10.1016/s0020-7292(00)00341-6

[47] D. Young , K. Fitzgerald , L. Laursen , and A. K. Whitaker , “Comparison of Vaginal and Buccal Misoprostol After Mifepristone for Medication Abortion Through 70 Days of Gestation: A Retrospective Chart Review,” Contraception (Stoneham) 115 (2022): 62–66. 10.1016/j.contraception.2022.06.012 35772525

[48] H.‐K. Au , C.‐F. Liu , and L.‐W. Chien , “Clinical Factors Associated With Subsequent Surgical Intervention in Women Undergoing Early Medical Termination of Viable or Non‐Viable Pregnancies,” Frontiers in Medicine 11 (2024): 1188629. 38737765 10.3389/fmed.2024.1188629 PMC11082305

[49] N. K. F. Abou Elela , “Vaginal Misoprostol Safety and Efficacy in Second Trimester Pregnancy Termination in Women With a Previous Cesarean Section,” Egyptian Journal of Hospital Medicine 88, no. 1 (2022): 3112–3116.

[50] E. Aydin and O. Ozyuncu , “Low‐Dose Misoprostol for Second Trimester Pregnancy Termination in Women With a Prior Caesarean Delivery,” Journal of Clinical and Diagnostic Research 13, no. 11 (2019): QC05–QC07, 10.7860/jcdr/2019/28209.13287.

[51] R. Bahar , H. Alexandroni , G. Karavani , R. Gilad , and A. Benshushan , “Safety of Medical Second Trimester Abortions for Women With Prior Cesarean Sections,” Archives of Gynecology and Obstetrics 303, no. 5 (2021): 1217–1222. 33386956 10.1007/s00404-020-05904-9

[52] J. K. Basu and D. Basu , “The Management of Failed Second‐Trimester Termination of Pregnancy,” Contraception 80, no. 2 (2009): 170–173. 19631793 10.1016/j.contraception.2009.01.015

[53] N. Bhattacharjee , R. P. Ganguly , and S. P. Saha , “Misoprostol for Termination of Mid‐Trimester Post‐Caesarean Pregnancy,” Australian and New Zealand Journal of Obstetrics and Gynaecology 47, no. 1 (2007): 23–25. 17261095 10.1111/j.1479-828X.2006.00673.x

[54] K. Bhuvaneswari , “Study on Midtrimester Termination of Pregnancy in Previous Caesarean Section: Comparison Between Intracervical Foley With Misoprostol and Mifepristone With Misoprostol: Madras Medical College, Chennai,” 2020.

[55] J. F. Brouns , M. van Wely , M. P. Burger , and W. J. van Wijngaarden , “Comparison of Two Dose Regimens of Misoprostol for Second‐Trimester Pregnancy Termination,” Contraception 82, no. 3 (2010): 266–275. 20705156 10.1016/j.contraception.2010.03.006

[56] C. Cetin , S. Buyukkurt , G. Seydaoglu , B. Kahveci , C. Soysal , and F. T. Ozgunen , “Comparison of Two Misoprostol Regimens for Mid‐Trimester Pregnancy Terminations After FIGOs Misoprostol Dosage Recommendation in 2012,” Journal of Maternal‐Fetal and Neonatal Medicine 29, no. 8 (2016): 1314–1317. 26067264 10.3109/14767058.2015.1046831

[57] Y. Chen , L. Zhang , Y. Xu , and P. Yang , “Clinical Analysis of the Regimens for Terminating the Second‐Trimester Pregnancy in Cesarean Section Women,” Journal of Maternal‐Fetal & Neonatal Medicine 36, no. 2 (2023): 2249187. 37654101 10.1080/14767058.2023.2249187

[58] N. Choudhary , R. Bagga , A. Raveendran , S. C. Saha , and L. K. Dhaliwal , “Second Trimester Abortion in Women With and Without Previous Uterine Scar: Eleven Years Experience From a Developing Country,” European Journal of Contraception & Reproductive Health Care 16, no. 5 (2011): 378–386. 21929363 10.3109/13625187.2011.599453

[59] A. Daponte , G. Nzewenga , K. D. Dimopoulos , and F. Guidozzi , “The Use of Vaginal Misoprostol for Second‐Trimester Pregnancy Termination in Women With Previous Single Cesarean Section,” Contraception 74, no. 4 (2006): 324–327. 16982234 10.1016/j.contraception.2006.03.023

[60] A. Daponte , G. Nzewenga , K. D. Dimopoulos , and F. Guidozzi , “Pregnancy Termination Using Vaginal Misoprostol in Women With More Than One Caesarean Section,” Journal of Obstetrics and Gynaecology 27, no. 6 (2007): 597–600. 17896259 10.1080/01443610701497561

[61] G. J. Daskalakis , S. A. Mesogitis , N. E. Papantoniou , G. G. Moulopoulos , A. A. Papapanagiotou , and A. J. Antsaklis , “Misoprostol for Second Trimester Pregnancy Termination in Women With Prior Caesarean Section,” BJOG: An International Journal of Obstetrics and Gynaecology 112, no. 1 (2005): 97–99. 10.1111/j.1471-0528.2004.00285.x 15663405

[62] A. Davey , “Oral Mifepristone 600 Mg and Vaginal Gemeprost for Mid‐Trimester Induction of Abortion: An Open Multicenter Study,” Contraception 56, no. 6 (1997): 361–366. 9494769 10.1016/s0010-7824(97)00184-4

[63] J. E. Dickinson , “Misoprostol for Second‐Trimester Pregnancy Termination in Women With a Prior Cesarean Delivery,” Obstetrics and Gynecology 105, no. 2 (2005): 352–356. 15684164 10.1097/01.AOG.0000151996.16422.88

[64] J. E. Dickinson , P. Brownell , K. McGinnis , and E. A. Nathan , “Mifepristone and Second Trimester Pregnancy Termination for Fetal Abnormality in Western Australia: Worth the Effort,” Australian and New Zealand Journal of Obstetrics and Gynaecology 50, no. 1 (2010): 60–64. 20218999 10.1111/j.1479-828X.2009.01117.x

[65] J. E. Dickinson and D. A. Doherty , “Mifepristone Priming and Subsequent Misoprostol for Second Trimester Medical Abortion in Women With Previous Caesarean Delivery,” Australian and New Zealand Journal of Obstetrics and Gynaecology 63, no. 3 (2023): 301–307. 36789734 10.1111/ajo.13653

[66] C. M. Domröse , A. Geipel , C. Berg , H. Lorenzen , U. Gembruch , and A. Willruth , “Second‐ and Third‐Trimester Termination of Pregnancy in Women With Uterine Scar ‐ A Retrospective Analysis of 111 Gemeprost‐Induced Terminations of Pregnancy After Previous Cesarean Delivery,” Contraception 85, no. 6 (2012): 589–594. 22079607 10.1016/j.contraception.2011.10.005

[67] A. N. Elasy , M. A. M. Ibrahem , L. L. Elhawy , and B. M. Hamed , “Vaginal Misoprostol Versus Combined Intracervical Foley's Catheter and Oxytocin Infusion for Second Trimester Pregnancy Termination in Women With Previous Caesarean Sections: A Randomised Control Trial,” Journal of Obstetrics and Gynaecology 42, no. 7 (2022): 2962–2969. 36149628 10.1080/01443615.2022.2118572

[68] T. H. El‐Sayed , A. A. Abulnour , A. M. Mohamed Abdelsalam , and H. A. Abdelbasset , “The Effect of Dilapan‐s vs. Misoprostol as a Cervical Ripening Agent in 2nd Trimesteric Abortion With Scarred Uterus,” Ginekologia i Poloznictwo 2, no. 66 (2023): 1–5.

[69] I. A. E. El Sharkwy , M. L. Elsayed , M. A. Ahmed , and A. A. A. Alnemer , “Low‐Dose Vaginal Misoprostol With or Without Foley Catheter for Late Second‐Trimester Pregnancy Termination in Women With Previous Multiple Cesarean Sections,” Journal of Maternal‐Fetal & Neonatal Medicine 32, no. 22 (2019): 3703–3707. 29742942 10.1080/14767058.2018.1470236

[70] Ö. Ercan , B. Köstü , A. Özer , S. Serin , and M. Bakacak , “Misoprostol Versus Misoprostol and Foley Catheter Combination in 2nd Trimester Pregnancy Terminations,” Journal of Maternal‐Fetal & Neonatal Medicine 29, no. 17 (2016): 2810–2812. 26452400 10.3109/14767058.2015.1105950

[71] A. Erturk , B. T. Karapinar , F. N. Tasgoz , B. Dundar , and N. Kender Erturk , “The Safety of Misoprostol Alone Use for Second‐Trimester Termination of Pregnancy in Women With Previous Caesarean Deliveries,” European Journal of Contraception & Reproductive Health Care 27, no. 6 (2022): 473–477. 36062521 10.1080/13625187.2022.2115836

[72] M. Fawzy and E. S. Abdel‐Hady , “Midtrimester Abortion Using Vaginal Misoprostol for Women With Three or More Prior Cesarean Deliveries,” International Journal of Gynecology & Obstetrics 110, no. 1 (2010): 50–52, 10.1016/j.ijgo.2010.02.008. 20362989

[73] G. Garofalo , A. Garofalo , O. Sochirca , et al., “Maternal Outcomes in First and Second Trimester Termination of Pregnancy: Which Are the Risk Factors?,” Journal of Perinatal Medicine 46, no. 4 (2018): 373–378. 29055174 10.1515/jpm-2017-0106

[74] O. Gómez , A. Borrás , A. Rabanal , et al., “Mifepristone–Misoprostol Midtrimester Abortion: Impact of Gestational Age on the Induction‐To‐Abortion Interval,” Contraception (Stoneham) 81, no. 2 (2010): 97–101. 10.1016/j.contraception.2009.10.001 20103444

[75] U. K. Güleç , I. F. Urunsak , E. Eser , et al., “Misoprostol for Midtrimester Termination of Pregnancy in Women With 1 or More Prior Cesarean Deliveries,” International Journal of Gynecology & Obstetrics 120, no. 1 (2013): 85–87, 10.1016/j.ijgo.2012.08.013. 23195293

[76] A. Henkel , K. Lerma , P. D. Blumenthal , and K. A. Shaw , “Evaluation of Shorter Mifepristone to Misoprostol Intervals for Second Trimester Medical Abortion: A Retrospective Cohort Study,” Contraception 102, no. 5 (2020): 327–331. 32592800 10.1016/j.contraception.2020.06.009

[77] Y. Herabutya , B. Chanarachakul , and P. Punyavachira , “Induction of Labor With Vaginal Misoprostol for Second Trimester Termination of Pregnancy in the Scarred Uterus,” International Journal of Gynecology & Obstetrics 83, no. 3 (2003): 293–297. 14643040 10.1016/s0020-7292(03)00312-6

[78] S. Hou , L. Zhang , Q. Chen , A. Fang , and L. Cheng , “One‐ and Two‐Day Mifepristone–Misoprostol Intervals for Second Trimester Termination of Pregnancy Between 13 and 16 Weeks of Gestation,” International Journal of Gynecology & Obstetrics 111, no. 2 (2010): 126–130. 20705290 10.1016/j.ijgo.2010.06.008

[79] L. Jacques , M. Heinlein , J. Ralph , et al., “Complication Rates of Dilation and Evacuation and Labor Induction in Second‐Trimester Abortion for Fetal Indications: A Retrospective Cohort Study,” Contraception 102, no. 2 (2020): 83–86. 32360665 10.1016/j.contraception.2020.04.018

[80] M. Jamali , M. Bakhtiyari , F. Arab , and M. Mirzamoradi , “Misoprostol Complications in Second‐Trimester Termination of Pregnancy Among Women With a History of More Than One Cesarean Section,” Obstetrics and Gynecology Science 63, no. 3 (2020): 323–329. 32489977 10.5468/ogs.2020.63.3.323 PMC7231932

[81] N. Kapp , L. Borgatta , P. Stubblefield , O. Vragovic , and N. Moreno , “Mifepristone in Second‐Trimester Medical Abortion: A Randomized Controlled Trial,” Obstetrics and Gynecology (New York, N.Y.) 110, no. 6 (2007): 1304–1310. 10.1097/01.AOG.0000289577.32274.a5 18055725

[82] J. Kiley , A. Turner , C. Nosal , M. Beestrum , and J. Dungan , “Labour Induction for Termination of Pregnancy With Severe Fetal Anomalies After 24 Weeks' Gestation: A Case Series and Systematic Review of the Literature,” European Journal of Contraception & Reproductive Health Care 27, no. 6 (2022): 486–493. 35899830 10.1080/13625187.2022.2102604

[83] D. Seow Choon K , E. Chaw T , H. Chang Qi QL , et al., “Incidence and Contributing Factors for Uterine Rupture in Patients Undergoing Second Trimester Termination of Pregnancy in a Large Tertiary Hospital—a 10‐Year Case Series,” European Journal of Obstetrics, Gynecology, and Reproductive Biology 227 (2018): 8–12, 10.1016/j.ejogrb.2018.05.016. 29860060

[84] K. Latta , E. Barker , P. Kendall , et al., “Complications of Second Trimester Induction for Abortion or Fetal Demise for Patients With and Without Prior Cesarean Delivery,” Contraception 117 (2023): 55–60. 35760083 10.1016/j.contraception.2022.06.011

[85] N. F. Liaquat , I. Javed , S. Shuja , et al., “Therapeutic Termination of Second Trimester Pregnancies With Low Dose Misoprostol,” Journal of the College of Physicians and Surgeons–Pakistan 16, no. 7 (2006): 464–467. 16827957

[86] E. Marinoni , M. Santoro , M. P. Vitagliano , A. Patella , E. V. Cosmi , and R. Di Iorio , “Intravaginal Gemeprost and Second‐Trimester Pregnancy Termination in the Scarred Uterus,” International Journal of Gynecology & Obstetrics 97, no. 1 (2007): 35–39. 17320086 10.1016/j.ijgo.2006.12.013

[87] N. M. Masse , K. Kuchta , B. A. Plunkett , and D. W. Ouyang , “Complications Associated With Second Trimester Inductions of Labor Requiring Greater Than Five Doses of Misoprostol,” Contraception (Stoneham) 101, no. 1 (2020): 53–55. 10.1016/j.contraception.2019.09.004 31655074

[88] C. Mazouni , M. Provensal , G. Porcu , et al., “Termination of Pregnancy in Patients With Previous Cesarean Section,” Contraception 73, no. 3 (2006): 244–248. 16472563 10.1016/j.contraception.2005.09.007

[89] A. Meaidi , J. S. Friedrich , and Ø. Lidegaard , “Risk of Surgical Evacuation and Risk of Major Surgery Following Second‐Trimester Medical Abortion in Denmark: A Nationwide Cohort Study,” Contraception 102, no. 3 (2020): 201–206, 10.1016/j.contraception.2020.04.017. 32511945

[90] I. Mobusher , “Misoprostol for Second Trimester Pregnancy Termination in Women With Prior Caesarean Section,” Pakistan Journal of Medical and Health Sciences 7, no. 1 (2013): 130–132.

[91] I. Morra , C. Ferrara , G. Sglavo , et al., “Incidence of Uterine Rupture in Second‐Trimester Abortion With Gemeprost Alone Compared to Mifepristone and Gemeprost,” Contraception 99, no. 3 (2019): 152–154. 30468720 10.1016/j.contraception.2018.11.004

[92] S. I. Munir , S. Ishtiaq , S. Shafiq , L. Javed , and T. Rana , “Mid Trimester Termination of Pregnancy in Women With Previous Caesarean Section: A Comparison of Misoprostol, PGF2α and Intra‐Cervical Foley's Catheter Traction,” Pakistan Journal of Medical and Health Sciences 8, no. 3 (2014): 554–558.

[93] A. H. Naguib , H. M. Morsi , T. F. Borg , S. T. Fayed , and H. M. Hemeda , “Vaginal Misoprostol for Second‐Trimester Pregnancy Termination After One Previous Cesarean Delivery,” International Journal of Gynecology & Obstetrics 108, no. 1 (2010): 48–51. 19781700 10.1016/j.ijgo.2009.08.010

[94] M. Obata‐Yasuoka , H. Hamada , H. Watanabe , et al., “Midtrimester Termination of Pregnancy Using Gemeprost in Combination With Laminaria in Women Who Have Previously Undergone Cesarean Section,” Journal of Obstetrics and Gynaecology Research 35, no. 5 (2009): 901–905. 20149039 10.1111/j.1447-0756.2009.01044.x

[95] P. Peng , X. Y. Liu , L. Li , L. Jin , and W. L. Chen , “Clinical Analyses of 66 Cases of Mid‐Trimester Pregnancy Termination in Women With Prior Cesarean,” Chinese Medical Journal 128, no. 4 (2015): 450–454. 25673444 10.4103/0366-6999.151073 PMC4836245

[96] A. Petca , “Second Trimester Abortion: Mifepristone Plus Misoprostol vs. Misoprostol Plus Oxytocin,” Farmácia 67, no. 5 (2019): 850–856, 10.31925/farmacia.2019.5.14.

[97] S. Pongsatha and T. Tongsong , “Outcomes of Pregnancy Termination by Misoprostol at 14‐32 Weeks of Gestation: A 10‐Year‐Experience,” Journal of the Medical Association of Thailand 94, no. 8 (2011): 897–901. 21863669

[98] S. Pongsatha , N. Suntornlimsiri , and T. Tongsong , “Comparing the Outcomes of Termination of Second Trimester Pregnancy With a Live Fetus Using Intravaginal Misoprostol Between Women With and Without Previous Cesarean Section,” BMC Pregnancy and Childbirth 24, no. 1 (2024): 274. 38609883 10.1186/s12884-024-06442-x PMC11015687

[99] S. A. Pourhoseini , S. Niroumand , A. Akbari , et al., “Comparing the Effects of Misoprostol/Letrozole and Misoprostol/Placebo on Medical Abortion Success Rate: A Randomized Clinical Trial,” Shiraz e‐Medical Journal 24, no. 4 (2023): e131460.

[100] E. Reehan , S. J. Abid , S. Sarsam , T. N. Abdulla , and Z. Al‐Attar , “Misoprostol and Mid Trimester Termination of Pregnancy in Patients With Two Previous Scars and More at Elwiya Maternity Teaching Hospital,” Revista Brasileira de Saude Materno Infantil 24 (2024): e20220357.

[101] T. Reischer , I. Limbach , A. Catic , K. Niedermaier , V. Falcone , and G. Yerlikaya‐Schatten , “Factors Influencing the Duration of Termination of Pregnancy for Fetal Anomaly With Mifepristone in Combination With Misoprostol,” Journal of Clinical Medicine 12, no. 3 (2023): 869. 36769518 10.3390/jcm12030869 PMC9918131

[102] M. Scioscia , G. Pontrelli , A. Vimercati , S. Santamato , and L. Selvaggi , “A Short‐Scheme Protocol of Gemeprost for Midtrimester Termination of Pregnancy With Uterine Scar,” Contraception (Stoneham) 71, no. 3 (2005): 193–196. 10.1016/j.contraception.2004.10.012 15722069

[103] A. G. Shammas and M. D. Momani , “Misoprostol for Termination of Second Trimester Pregnancy in a Scarred Uterus,” Saudi Medical Journal 27, no. 8 (2006): 1173–1176. 16883447

[104] U. Shantikumar , R. Bagga , J. Kalra , et al., “Second‐Trimester Medical Abortion With Misoprostol Preceded by Two Sequential Doses of Mifepristone: An Observational Study,” Journal of Obstetrics and Gynaecology of India 72, no. Suppl 1 (2022): 26–35. 10.1007/s13224-021-01521-x PMC9343499 35928056

[105] J. Sharma , S. Tiwari , M. Pokhrel , and L. Lama , “Medical Induction for Mid Trimester Abortion: A Hospital‐Based Descriptive Cross‐Sectional Study,” Journal of Nepal Medical Association 58, no. 230 (2020): 794–797. 10.31729/jnma.5502 PMC7654484 34504357

[106] R. L. Shay , L. S. Benson , E. M. Lokken , and E. A. Micks , “Same‐Day Mifepristone Prior to Second‐Trimester Induction Termination With Misoprostol: A Retrospective Cohort Study,” Contraception (Stoneham) 107 (2022): 29–35. 10.1016/j.contraception.2021.09.006 34529952

[107] B. Stewart , S. C. Kane , and J. Unterscheider , “Medical Termination of Pregnancy for Fetal Anomaly at or Beyond 20 Weeks' Gestation‐What Are the Maternal Risks?,” Prenatal Diagnosis 42, no. 12 (2022): 1562–1570. 36156270 10.1002/pd.6241

[108] E. Tarim , E. Kilicdag , T. Bagis , A. Ilgin , and F. Yanik , “Second‐Trimester Pregnancy Termination With Oral Misoprostol in Women Who Have Had One Cesarean Section,” International Journal of Gynecology & Obstetrics 90, no. 1 (2005): 84–85. 15992552 10.1016/j.ijgo.2004.12.047

[109] M. Cuellar Torriente , W. J. Steinberg , and G. Joubert , “Misoprostol Use for Second‐Trimester Termination of Pregnancy Among Women With One or More Previous Cesarean Deliveries,” International Journal of Gynecology & Obstetrics 138, no. 1 (2017): 23–27. 28378361 10.1002/ijgo.12168

[110] A. Turgut , A. Özler , N. Y. Görük , T. Karaçor , and A. Yalinkaya , “Misoprostol‐Induced Termination of Second‐Trimester Pregnancy in Women With a History of Cesarean Section: A Retrospective Analysis of 56 Cases,” Ginekologia Polska 84, no. 4 (2013): 277–280. 23700860

[111] L. J. van Bogaert , “Termination of Pregnancy With Misoprostol in the Scarred Uterus,” International Journal of Gynecology & Obstetrics 100, no. 1 (2007): 80–81. 17888919 10.1016/j.ijgo.2007.05.043

[112] M. Velipasaoglu , C. Y. Ozdemir , B. Ozek , R. Ayaz , and H. M. Tanir , “Sequential Use of Foley Catheter With Misoprostol for Second Trimester Pregnancy Termination in Women With and Without Caesarean Scars: A Prospective Cohort Study,” Journal of Maternal‐Fetal & Neonatal Medicine 31, no. 5 (2018): 677–681. 28282779 10.1080/14767058.2017.1293037

[113] S. Vlad , I. Boucoiran , E. R. St‐Pierre , and E. Ferreira , “Mifepristone‐Misoprostol Use for Second‐ and Third‐Trimester Medical Termination of Pregnancy in a Canadian Tertiary Care Centre,” Journal of Obstetrics and Gynaecology Canada 44, no. 6 (2022): 683–689. 35114381 10.1016/j.jogc.2021.12.010

[114] M. Anant , A. Paswan , and C. Jyoti , “Cesarean Scar Ectopic Pregnancy: The Lurking Danger in Post Cesarean Failed Medical Abortion,” Journal of Family & Reproductive Health 13, no. 4 (2019): 223–227. 32518574 PMC7264869

[115] J. E. Dickinson and S. F. Evans , “The Optimization of Intravaginal Misoprostol Dosing Schedules in Second‐Trimester Pregnancy Termination,” American Journal of Obstetrics and Gynecology 186, no. 3 (2002): 470–474. 11904609 10.1067/mob.2002.121085

[116] J. E. Dickinson and D. A. Doherty , “Maternal Complications Associated With Second Trimester Medical Abortion Using Mifepristone Priming and Subsequent Misoprostol,” Contraception 125 (2023): 110080. 37245784 10.1016/j.contraception.2023.110080

[117] A. Meaidi , S. Friedrich , T. A. Gerds , and O. Lidegaard , “Risk Factors for Surgical Intervention of Early Medical Abortion,” American Journal of Obstetrics and Gynecology 220, no. 5 (2019): 478.e1–478.e15. 10.1016/j.ajog.2019.02.014 30763542

[118] S. J. Chapman , M. Crispens , J. Owen , and K. Savage , “Complications of Midtrimester Pregnancy Termination: The Effect of Prior Cesarean Delivery,” American Journal of Obstetrics and Gynecology 175, no. 4 Pt 1 (1996): 889–892, 10.1016/s0002-9378(96)80019-6. 8885742

[119] J. L. C. Esteve , F. G. Gallego , M. P. Llorente , et al., “Late Second‐Trimester Abortions Induced With Mifepristone, Misoprostol and Oxytocin: A Report of 428 Consecutive Cases,” Contraception 78, no. 1 (2008): 52–60. 18555818 10.1016/j.contraception.2008.02.016

[120] Ö. Dural , C. Y , G. Yildirim , M. Bestel , S. Tekeli , and H. Aslan , “Comparison of Use of Low Dose Vaginal Misoprostol for Second and Early Third Trimester Pregnancy Termination in Women With Prior Caesarean and Unscarred Uteri,” Journal of Istanbul Faculty of Medicine 79, no. 2 (2016): 72–78.

[121] K. A. Uribe , A. Q. Nguyen , A. E. Burke , and C. Johnson , “Second Trimester Induction in the Setting of a Prior Cesarean Delivery [9R],” Obstetrics and Gynecology (New York 1953) 133, no. Suppl 1 (2019): 193S–194S.

[122] P. Boulot , M. Hoffet , B. Bachelard , et al., “Late Vaginal Induced Abortion After a Previous Cesarean Birth: Potential for Uterine Rupture,” Gynecologic and Obstetric Investigation 36, no. 2 (1993): 87–90. 8225053 10.1159/000292602

[123] M. Cayrac , J.‐L. Faillie , A. Flandrin , and P. Boulot , “Second‐and Third‐Trimester Management of Medical Termination of Pregnancy and Fetal Death In Utero After Prior Caesarean Section,” European Journal of Obstetrics & Gynecology and Reproductive Biology 157, no. 2 (2011): 145–149. 21511389 10.1016/j.ejogrb.2011.03.013

[124] M. Driessen , M. Dommergues , L. Mandelbrot , I. Durand‐Zaleski , N. Boudjema , and J. Nizard , “OP26.02: Second and Third Trimester Termination of Pregnancy for Fetal Anomaly: Impact of a Previous Caesarean Section on Maternal Morbidity,” Ultrasound in Obstetrics & Gynecology 38, no. S1 (2011): 131.

[125] M. A. De Boer , N. Van Gemund , S. A. Scherjon , and H. H. H. Kanhai , “Low Dose Sulprostone for Termination of Second and Third Trimester Pregnancies,” European Journal of Obstetrics & Gynecology and Reproductive Biology 99, no. 2 (2001): 244–248. 11788180 10.1016/s0301-2115(01)00406-7

[126] P. A. Le Roux , G. S. Pahal , L. Hoffman , R. Nooh , H. El‐Refaey , and C. H. Rodeck , “Second Trimester Termination of Pregnancy for Fetal Anomaly or Death: Comparing Mifepristone/Misoprostol to Gemeprost,” European Journal of Obstetrics & Gynecology and Reproductive Biology 95, no. 1 (2001): 52–54. 11267720 10.1016/s0301-2115(00)00365-1

[127] B. Iftikhar , R. Khanum , N. A. Akram , S. Nasreen , F. Ibrar , and A. Fatah , “A Comparison of Complications in Previous Caesarean With Non Caesarean Cases Undergoing Misoprostol Induced Mid Trimester Abortions,” Pakistan Armed Forces Medical Journal 69, no. 3 (2019): 528–533.

[128] M. Hoopmann , J. Hirneth , J. Pauluschke‐Fröhlich , et al., “Influence of Mifepristone in Induction Time for Terminations in the Second and Third Trimester,” Geburtshilfe und Frauenheilkunde 74, no. 4 (2014): 350–354. 25076791 10.1055/s-0033-1360361 PMC4078152

[129] N. Wagner , H. Abele , M. Hoopmann , et al., “Factors Influencing the Duration of Late First and Second‐Trimester Termination of Pregnancy With Prostaglandin Derivates,” European Journal of Obstetrics, Gynecology, and Reproductive Biology 155, no. 1 (2011): 75–78. 21112135 10.1016/j.ejogrb.2010.10.019

[130] T. Spingler , J. Sonek , M. Hoopmann , N. Prodan , H. Abele , and K. O. Kagan , “Complication Rate After Termination of Pregnancy for Fetal Defects,” Ultrasound in Obstetrics & Gynecology 62, no. 1 (2023): 88–93. 36609996 10.1002/uog.26157

[131] N. Bhattacharjee , S. C. Biswas , S. Mukhopadhyay , S. P. Saha , R. P. Ganguly , and K. K. Patra , “Safety and Efficacy of Misoprostol for Termation of 1st and 2nd Trimester Post Cesarean Pregnancy,” Journal International Medical Sciences Academy 20, no. 2 (2007): 135–136.

[132] N. Anderson , C. Dehlendorf , R. Ali , J. Steinauer , and E. S. Lichtenberg , “Does a History of Prior Uterine Scarring Increase the Likelihood of Intervention Among Women Undergoing Medication Abortion?,” Contraception 90, no. 3 (2014): 303–304.

[133] C. E. Dehlendorf , E. E. Fox , R. F. Ali , N. C. Anderson , R. D. Reed , and E. S. Lichtenberg , “Medication Abortion Failure in Women With and Without Previous Cesarean Delivery,” Contraception 92, no. 5 (2015): 463–468. 26226101 10.1016/j.contraception.2015.07.011

[134] M. Mentula and O. Heikinheimo , “Risk Factors of Surgical Evacuation Following Second‐Trimester Medical Termination of Pregnancy,” Contraception 86, no. 2 (2012): 141–146. 22240172 10.1016/j.contraception.2011.11.070

[135] V. Nair , R. Mikkere , and K. Ojha , “P04.02: Medical Termination of Pregnancy After Caesarean Section: How Safe and Effective Is It?,” Ultrasound in Obstetrics & Gynecology 40, no. S1 (2012): 182.

[136] M. Odeh , R. Tendler , V. Sosnovsky , M. Kais , E. Ophir , and J. Bornstein , “The Effect of Parity and Gravidity on the Outcome of Medical Termination of Pregnancy,” Israel Medical Association Journal 12, no. 10 (2010): 606–608. 21090516

[137] M. F. Reeves , J. A. Monmaney , and M. D. Creinin , “Predictors of Uterine Evacuation Following Early Medical Abortion With Mifepristone and Misoprostol,” Contraception 93, no. 2 (2016): 119–125. 26285178 10.1016/j.contraception.2015.08.010

[138] H. G. Çelik , S. Y. Semerci , G. Yildirim , and M. Çetinkaya , “Iniencephaly: A Rare Congenital Anomaly Reaching the Term,” Case Reports in Perinatal Medicine 6 (2017): 1–3.

[139] H. M. Chang , C. J. Shen , C. Y. Lin , and E. M. Tsai , “Uterine Perforation and Bowel Incarceration Following Surgical Abortion During the First Trimester,” Taiwanese Journal of Obstetrics & Gynecology 47, no. 4 (2008): 448–450. 19126515 10.1016/S1028-4559(09)60016-4

[140] B. B. Forster , C. M. Siu , J. B. Murray , and M. H. Chung , “Transabdominal and Transvaginal Ultrasonography of Uterine Perforation Following Suction Curettage,” Canadian Association of Radiologists Journal 40, no. 6 (1989): 318–319. 2688839

[141] I. Ben‐Ami , D. Schneider , R. Svirsky , N. Smorgick , M. Pansky , and R. Halperin , “Safety of Late Second‐Trimester Pregnancy Termination by Laminaria Dilatation and Evacuation in Patients With Previous Multiple Cesarean Sections,” American Journal of Obstetrics & Gynecology 201, no. 2 (2009): 154.e1–154.e5. 10.1016/j.ajog.2009.04.029 19539892

[142] R. Chodankar , J. Gupta , D. Gdovinova , et al., “Synthetic Osmotic Dilators for Cervical Preparation Prior to Abortion—An International Multicentre Observational Study,” European Journal of Obstetrics, Gynecology, and Reproductive Biology 228 (2018): 249–254. 30032065 10.1016/j.ejogrb.2018.07.013

[143] W. M. Hern , “Misoprostol as an Adjunctive Medication in Late Surgical Abortion,” International Journal of Gynecology & Obstetrics 88, no. 3 (2005): 327–328. 15733894 10.1016/j.ijgo.2004.12.008

[144] M. D. Limongelli , F. Belletti , A. T. Cecere , L. Oliva , and G. Carlomagno , “Vaginal Misoprostol as a Cervical Ripening Agent Prior to Suction Curettage for First‐Trimester Termination of Pregnancy,” Italian Journal of Gynaecology and Obstetrics 16, no. 1 (2004): 32–34.

[145] R. Lyus , P. A. Lohr , J. Taylor , and C. Morroni , “Outcomes With Same‐Day Cervical Preparation With Dilapan‐S Osmotic Dilators and Vaginal Misoprostol Before Dilatation and Evacuation at 18 to 21+6 Weeks' Gestation,” Contraception (Stoneham) 87, no. 1 (2013): 71–75. 10.1016/j.contraception.2012.07.006 22898362

[146] A. Patel , E. Talmont , J. Morfesis , et al., “Adequacy and Safety of Buccal Misoprostol for Cervical Preparation Prior to Termination of Second‐Trimester Pregnancy,” Contraception (Stoneham) 73, no. 4 (2006): 420–430. 10.1016/j.contraception.2005.10.004 16531179

[147] S. Ramesh , A. Roston , L. Zimmerman , A. Patel , S. Lichtenberg , and J. Chor , “One‐Hour Buccal Misoprostol Compared With Osmotic Dilators for Cervical Preparation in Early Surgical Abortion,” Obstetrics and Gynecology 123 (2014): 108S–110S.

[148] S. J. Lambert , B. Lunde , L. Porsch , et al., “Adjuvant Misoprostol or Mifepristone for Cervical Preparation With Osmotic Dilators Before Dilation and Evacuation,” Contraception 132 (2024): 110364. 38218312 10.1016/j.contraception.2024.110364

[149] A. C. Frick , E. A. Drey , J. T. Diedrich , and J. E. Steinauer , “Effect of Prior Cesarean Delivery on Risk of Second‐Trimester Surgical Abortion Complications,” Obstetrics and Gynecology 115, no. 4 (2010): 760–764. 20308836 10.1097/AOG.0b013e3181d43f42

[150] M. Guiahi , G. Schiller , J. Sheeder , and S. Teal , “Safety of First‐Trimester Uterine Evacuation in the Outpatient Setting for Women With Common Chronic Conditions,” Contraception 92, no. 5 (2015): 453–457. 26197262 10.1016/j.contraception.2015.07.005

[151] L. Lederle , J. E. Steinauer , A. Montgomery , S. Aksel , E. A. Drey , and J. L. Kerns , “Obesity as a Risk Factor for Complications After Second‐Trimester Abortion by Dilation and Evacuation,” Obstetrics & Gynecology 126, no. 3 (2015): 585–592, 10.1097/AOG.0000000000001006. 26244536 PMC4545380

[152] L. A. Murphy , L. L. Thornburg , J. C. Glantz , E. C. Wasserman , N. L. Stanwood , and S. J. Betstadt , “Complications of Surgical Termination of Second‐Trimester Pregnancy in Obese Versus Nonobese Women,” Contraception 86, no. 4 (2012): 402–406. 22445440 10.1016/j.contraception.2012.02.006

[153] B. R. Pridmore and D. G. Chambers , “Uterine Perforation During Surgical Abortion: A Review of Diagnosis, Management and Prevention,” Australian and New Zealand Journal of Obstetrics and Gynaecology 39, no. 3 (1999): 349–353. 10554950 10.1111/j.1479-828x.1999.tb03413.x

[154] D. Schneider , I. Bukovsky , and E. Caspi , “Safety of Midtrimester Pregnancy Termination by Laminaria and Evacuation in Patients With Previous Cesarean Section,” American Journal of Obstetrics and Gynecology 171, no. 2 (1994): 554–557. 8059841 10.1016/0002-9378(94)90299-2

[155] K. S. Mark , B. Bragg , T. Talaie , K. Chawla , L. Murphy , and M. Terplan , “Risk of Complication During Surgical Abortion in Obese Women,” American Journal of Obstetrics and Gynecology 218, no. 2 (2018): 238.e1–238.e5. 10.1016/j.ajog.2017.10.018 29074080

[156] L. C. Wilson , L. A. Meyn , and M. D. Creinin , “Cervical Preparation for Surgical Abortion Between 12 and 18 Weeks of Gestation Using Vaginal Misoprostol and Dilapan‐S,” Contraception (Stoneham) 83, no. 6 (2011): 511–516. 10.1016/j.contraception.2010.10.004 21570547

[157] P. A. Lohr , J. H. Parsons , J. Taylor , and C. Morroni , “Outcomes of Dilation and Evacuation With and Without Feticide by Intra‐Cardiac Potassium Chloride Injection: A Service Evaluation,” Contraception 98, no. 2 (2018): 100–105. 10.1016/j.contraception.2018.04.010 29680767

[158] M. Ataei , F. Jalilvand , and M. Hashemnejad , “Successful Management of Non‐Diagnosed Placenta Percreta During Dilatation & Extraction Surgery,” International Journal of Pharmaceutical Research 10, no. 4 (2018): 879–881.

[159] P. Balkanli‐Kaplan , F. Gucer , F. Oz‐Puyan , and M. A. Yuce , “Placenta Percreta Diagnosed After First‐Trimester Pregnancy Termination: A Case Report,” Journal of Reproductive Medicine 51, no. 8 (2006): 662–664. 16967639

[160] J. Einenkel , P. Stumpp , S. Kösling , L.‐C. Horn , and M. Höckel , “A Misdiagnosed Case of Caesarean Scar Pregnancy,” Archives of Gynecology and Obstetrics 271, no. 2 (2005): 178–181. 15645280 10.1007/s00404-004-0683-1

[161] M. M. Hanstede , D. B. van't Hof , K. van Groningen , and I. M. de Graaf , “Severe Complication After Termination of a Second Trimester Cervical Pregnancy,” Fertility and Sterility 90, no. 5 (2008): 2009.e5–2009.e7. 10.1016/j.fertnstert.2008.03.014 18452917

[162] T. Orellana , A. Peters , and T. T. M. Lee , “Cesarean Section Scar Increta Following First Trimester Surgical Abortion: A Rare Phenomenon Requiring Hysterectomy,” Journal of Minimally Invasive Gynecology 27, no. 4 (2020): 800–802. 31472284 10.1016/j.jmig.2019.08.022

[163] R. Shojai , P. Roblin , and L. Boubli , “Failed Early Medical Abortion: Beware of the Uterine Scar!—Case Report,” European Journal of Contraception & Reproductive Health Care 17, no. 3 (2012): 237–239. 22497422 10.3109/13625187.2012.671386

[164] M. Kaba , C. Erkan , C. Sarica Mehmet , and A. Mayir Yeliz , “Emergency Hysterectomy After 2nd Trimester Abortion in a Patient With Placenta Accreta Spectrum Disorder Who Had Four Cesarean Deliveries,” Ceská Gynekologie 88, no. 2 (2023): 110–113, 10.48095/cccg2023110. 37130736

[165] K. E. Sharpless , I. I. Pappas , E. M. Dobrow , et al., “Severe Hemorrhage due to Acquired Uterine Arteriovenous Malformation/Fistula Following First‐Trimester Aspiration Abortion: A Case Report,” Case Reports in Women's Health 34 (2022): e00410. 10.1016/j.crwh.2022.e00410 PMC9035398 35479418

[166] E. J. Amarosa , M. Dighe , E. Y. Cheng , R. Garcia , and S. Delaney , “Management of Extensive Placenta Percreta With Induced Fetal Demise and Delayed Hysterectomy,” Case Reports in Perinatal Medicine 5, no. 1 (2016): 1–4.

[167] M. A. Bedaiwy , N. M. Grob , R. W. Redline , J. Pinkerton , L. K. Perriera , and N. Lazebnik , “Gravid Hysterectomy Following History of Recurrent Ruptured Uterus: Case Report,” Journal of Obstetrics and Gynaecology Research 37, no. 10 (2011): 1497–1502. 21599800 10.1111/j.1447-0756.2011.01540.x

[168] F. Chantraine , M. Nisolle , P. Petit , J. P. Schaaps , and J. M. Foidart , “Individual Decisions in Placenta Increta and Percreta: A Case Series,” Journal of Perinatal Medicine 40, no. 3 (2012): 265–270. 22505505 10.1515/jpm-2011-0156

[169] A. Kerr , D. Karlin , M. Mikhail , W. Rashbaum , and A. Anyaegbunam , “Intraoperative Embolization for Pelvic Hemorrhage Following Termination of Pregnancy,” American Journal of Perinatology 13, no. 3 (1996): 151–153. 8688105 10.1055/s-2007-994314

[170] S. Matsuzaki , S. Matsuzaki , Y. Ueda , et al., “A Case Report and Literature Review of Midtrimester Termination of Pregnancy Complicated by Placenta Previa and Placenta Accreta,” AJP Reports 5, no. 1 (2014): e6–e11. 26199801 10.1055/s-0034-1395992 PMC4502619

[171] K. Tocce , V. W. Thomas , S. Teal , K. Tocce , V. W. Thomas , and S. Teal , “Scheduled Hysterectomy for Second‐Trimester Abortion in a Patient With Placenta Accreta,” Obstetrics & Gynecology 113, no. 2 part 2 (2009): 568–570, 10.1097/aog.0b013e318194258c. 19155960

[172] R. Cui , M. Li , J. Lu , H. Bai , and Z. Zhang , “Management Strategies for Patients With Placenta Accreta Spectrum Disorders Who Underwent Pregnancy Termination in the Second Trimester: A Retrospective Study,” BMC Pregnancy and Childbirth 18, no. 1 (2018): 298. 29996794 10.1186/s12884-018-1935-6 PMC6042202

[173] Q. Hu , C. Li , L. Luo , et al., “Clinical Analysis of Second‐Trimester Pregnancy Termination After Previous Caesarean Delivery in 51 Patients With Placenta Previa and Placenta Accreta Spectrum: A Retrospective Study,” BMC Pregnancy and Childbirth 21, no. 1 (2021): 568, 10.1186/s12884-021-04017-8. 34407784 PMC8375210

[174] X. Liu , G. Fan , Z. Jin , et al., “Lower Uterine Segment Preganancy With Placenta Increta Complicating First Trimester Induced Abortion: Diagnosis and Conservative Management,” Chinese Medical Journal 116, no. 5 (2003): 695–698. 12875683

[175] J. Ou , P. Peng , L. Teng , C. Li , and X. Liu , “Management of Patients With Placenta Accreta Spectrum Disorders Who Underwent Pregnancy Terminations in the Second Trimester: A Retrospective Study,” European Journal of Obstetrics & Gynecology and Reproductive Biology 242 (2019): 109–113. 31580962 10.1016/j.ejogrb.2019.09.014

[176] W. K. Rashbaum , E. Jason Gates , J. Jones , B. Goldman , A. Morris , and W. D. Lyman , “Placenta Accreta Encountered During Dilation and Evacuation in the Second Trimester,” Obstetrics and Gynecology 85, no. 5 (1995): 701–703. 7724099 10.1016/0029-7844(95)00050-2

[177] H. J. van Beekhuizen , V. Stefanovic , A. Schwickert , et al., “A Multicenter Observational Survey of Management Strategies in 442 Pregnancies With Suspected Placenta Accreta Spectrum,” Acta Obstetricia et Gynecologica Scandinavica 100 (2021): 12–20. 33483943 10.1111/aogs.14096 PMC8048500

[178] L. Xie , Y. Wang , Y. C. Man , and F. Y. Luo , “Preliminary Experience in Uterine Artery Embolization for Second Trimester Pregnancy Induced Labor With Complete Placenta Previa, Placenta Implantation, and Pernicious Placenta Previa,” Clinical and Experimental Obstetrics & Gynecology 44, no. 1 (2017): 81–84. 29714871

[179] Q. Li , W. Zhang , C. Hu , et al., “Termination of a Second‐Trimester Pregnancy With Placenta Accreta Spectrum Disorder,” Libyan Journal of Medicine 18, no. 1 (2023): 2258669. 37722677 10.1080/19932820.2023.2258669 PMC10512921

[180] Q. Long , S. Wu , S. Du , R. Li , Y. Zhao , and F. Tang , “The Method for Termination of Mid‐Trimester Pregnancy With Placenta Previa: A Case Study,” Medicine 101, no. 31 (2022): e29908. 35945718 10.1097/MD.0000000000029908 PMC9351937

[181] M. Perdue , L. Felder , and V. Berghella , “First‐Trimester Uterine Rupture: A Case Report and Systematic Review of the Literature,” American Journal of Obstetrics and Gynecology 227 (2022): 209–217. 35487324 10.1016/j.ajog.2022.04.035

[182] I. E. M. D. Timor‐Tritsch and A. M. D. Monteagudo , “Unforeseen Consequences of the Increasing Rate of Cesarean Deliveries: Early Placenta Accreta and Cesarean Scar Pregnancy. A Review,” American Journal of Obstetrics and Gynecology 207, no. 1 (2012): 14–29, 10.1016/j.ajog.2012.03.007. 22516620

[183] F. Rahimi‐Sharbaf , A. Jamal , E. Mesdaghinia , M. Abedzadeh‐Kalahroudi , S. Niroomanesh , and F. Atoof , “Ultrasound Detection of Placenta Accreta in the First Trimester of Pregnancy,” Iranian Journal of Reproductive Medicine 12, no. 6 (2014): 421–426. 25071851 PMC4111891

[184] E. Jauniaux , A. Bhide , A. Kennedy , et al., “FIGO Consensus Guidelines on Placenta Accreta Spectrum Disorders: Prenatal Diagnosis and Screening,” International Journal of Gynecology & Obstetrics 140, no. 3 (2018): 274–280. 29405319 10.1002/ijgo.12408

[185] F. D'Antonio , I. E. Timor‐Tritsch , J. Palacios‐Jaraquemada , et al., “First‐Trimester Detection of Abnormally Invasive Placenta in High‐Risk Women: Systematic Review and Meta‐Analysis,” Ultrasound in Obstetrics & Gynecology 51, no. 2 (2018): 176–183. 28833750 10.1002/uog.18840

[186] S. Aragon , J. L. Carbonell , J. Mari , et al., “Oral Versus Vaginal Misoprostol for Cervical Priming in First‐Trimester Abortion: A Randomized Trial,” European Journal of Contraception & Reproductive Health Care 6, no. 3 (2001): 134–140, 10.1080/ejc.6.3.134.140. 11763976

[187] R.‐U. Khan , H. El‐Refaey , S. Sharma , D. Sooranna , and M. Stafford , “Oral, Rectal, and Vaginal Pharmacokinetics of Misoprostol,” Obstetrics and Gynecology (New York, N.Y.) 103, no. 5 (2004): 866–870. 10.1097/01.AOG.0000124783.38974.53 15121558

[188] Y. Cabrera , J. FernÁNdez‐Guisasola , P. Lobo , S. GÁmir , and J. ÁLvarez , “Comparison of Sublingual Versus Vaginal Misoprostol for Second‐Trimester Pregnancy Termination: A Meta‐Analysis,” Australian and New Zealand Journal of Obstetrics and Gynaecology 51, no. 2 (2011): 158–165. 21466519 10.1111/j.1479-828X.2010.01264.x

[189] I. Ben‐Ami , S. Stern , Z. Vaknin , N. Smorgick , D. Schneider , and R. Halperin , “Prevalence and Risk Factors of Inadequate Cervical Dilation Following Laminaria Insertion in Second‐Trimester Abortion—Case Control Study,” Contraception 91, no. 4 (2015): 308–312. 25575873 10.1016/j.contraception.2014.12.012

[190] R. A. Greene , C. Fitzpatrick , and M. J. Turner , “What Are the Maternal Implications of a Classical Caesarean Section?,” Journal of Obstetrics and Gynaecology 18, no. 4 (1998): 345–347. 15512105 10.1080/01443619867083

[191] Royal College of Obstetricians and Gynaecologists , “Birth After Previous Caesarean Birth: Greentop Guideline Number 45,” 2015.

[192] M. T. Y. Seto , S. F. Ngu , V. Y. T. Cheung , and T. C. Pun , “Second Trimester Medical Abortion in a Woman With Prior Classical Caesarean Section and a Uterine Leiomyoma‐A Case Report,” European Journal of Contraception & Reproductive Health Care 18, no. 5 (2013): 410–414. 23692523 10.3109/13625187.2013.797072

[193] J. Atad , A. Lissak , and I. Calderon , “Continuous Extraovular Prostaglandin F2 Alpha Instillation for Late Pregnancy Termination in Patients With Previous Cesarean Section Delivery,” International Journal of Gynecology & Obstetrics 24, no. 4 (1986): 315–319. 2878842 10.1016/0020-7292(86)90090-1

[194] S. G. Levrant and M. Wingate , “Midtrimester Uuterine Rupture. A Case Report,” Journal of Reproductive Medicine 41, no. 3 (1996): 186–190. 8778419

[195] S. Shapira , S. Goldberger , Y. Beyth , and M. D. Fejgin , “Induced Second Trimester Abortion by Extra‐Amniotic Prostaglandin Infusion in Patients With a Cesarean Scar: Is It Safe?,” Acta Obstetricia et Gynecologica Scandinavica 78, no. 6 (1999): 511–514. 10376860

[196] A. Debby , A. Golan , R. Sagiv , O. Sadan , and M. Glezerman , “Midtrimester Abortion in Patients With a Previous Uterine Scar,” European Journal of Obstetrics, Gynecology, and Reproductive Biology 109, no. 2 (2003): 177–180. 12860337 10.1016/s0301-2115(03)00121-0

[197] R. Malapati , G. Villaluna , and T. M. Nguyen , “Use of Misoprostol for Pregnancy Termination in Women With Prior Classical Cesarean Delivery: A Report of 3 Cases,” Journal of Reproductive Medicine 56, no. 1–2 (2011): 85–86. 21366135

[198] R. Pei , G. Wang , H. Wang , X. Huang , X. Yan , and X. Yang , “Efficacy and Safety of Prophylactic Uterine Artery Embolization in Pregnancy Termination With Placenta Previa,” Cardiovascular and Interventional Radiology 40, no. 3 (2017): 375–380. 27853824 10.1007/s00270-016-1507-y

[199] Z. Munn , T. H. Barker , S. Moola , et al., “Methodological Quality of Case Series Studies: An Introduction to the JBI Critical Appraisal Tool,” JBI Evidence Synthesis 18, no. 10 (2020): 2127–2133. 33038125 10.11124/JBISRIR-D-19-00099

[200] R. Morgan , A. Rooney , K. Taylor , et al., “Risk of Bias in Non‐Randomized Studies—of Exposure (ROBINS‐E). Launch version, 1 June 2022,” 1 October, 2024, https://www.riskofbias.info/welcome/robins‐e‐tool.

[201] L. A. Mcguinness and J. P. T. Higgins , “Risk‐of‐Bias VISualization (robvis): An R Package and Shiny Web App for Visualizing Risk‐of‐Bias Assessments,” Research Synthesis Methods 12, no. 1 (2020): 55–61. 32336025 10.1002/jrsm.1411

