# Supplementary material for: Induced Abortion After Previous Caesarean Section: A Scoping Review
Source: Aust N Z J Obstet Gynaecol. 2025 Apr 11;65(5):564–85. doi: 10.1111/ajo.70013 (PMC12723096; doi:10.1111/ajo.70013)
Supplement: Supplementary file 1 — Appendix S1 [file AJO-65-564-s004.docx]

**Appendix 1. Search Strategies**

MEDLINE (Ovid) search strategy for abortion outcomes and management after previous caesarean section*

| 1 | exp abortion, legal/ or Abortion, induced/ or Abortion, therapeutic/ |
| --- | --- |
| 2 | (abortion or "MTOP" or "D&E" or "dilation and evacuation" or "dilatation and evacuation" or (pregnan* adj3 terminat*)).tw. |
| 3 | Abortifacient agents/ or Abortifacient agents, nonsteroidal/ or Abortifacient agents, steroidal/ |
| 4 | 1 or 2 or 3 |
| 5 | exp caesarean section/ |
| 6 | (caesarean OR cesarean OR c-section).tw |
| 7 | 5 or 6 |
| 8 | (perforat* or rupture or hysterectom* or laparotom* or bleed* or "blood loss" or haemorrhage or hemorrhage or transfus* or dehiscence or complication* or death or safety or outcomes or safe or prevent* or manag* or ripen* or dilator or dilation or adverse or serious or major or sequelae or "retained products" or incomplete or laceration or accreta or previa or praevia).mp |
| 9 | 4 and 7 and 8 |
| 10 | Limit 9 to humans |
| 11 | Limit 10 to English language |

*Searched on 22/07/2024

CINAHL search strategy for abortion outcomes and management after previous caesarean section*

| S1 | MH “abortion, induced+” |
| --- | --- |
| S2 | AB abortion or "MTOP" or mifepristone or misoprostol or "D&E" or "dilation and evacuation" or "dilatation and evacuation" or (pregnan* N3 terminat*) |
| S3 | MH “abortifacient agents +” |
| S4 | S1 or S2 or S3 |
| S5 | MH “cesarean section+” |
| S6 | AB Caesarean OR caesarean OR c-section |
| S7 | S5 or S6 |
| S8 | TX perforat* or rupture or hysterectom* or laparotom* or bleed* or "blood loss" or haemorrhage or hemorrhage or transfus* or dehiscence or complication* or death or safety or outcomes or safe or prevent* or manag* or ripen* or dilator or dilation or adverse or serious or major or sequelae or "retained products" or incomplete or laceration or accreta or previa or praevia |
| S9 | S4 and S7 and S8 |
| S10 | Narrow S9 to English language |

*Searched on 22/07/2024

SCOPUS search strategy for abortion outcomes and management after previous caesarean section*

( ( TITLE-ABS-KEY ( "induced abortion"  OR  "medical abortion"  OR  "MTOP"  OR  "therapeutic abortion"  OR  "surgical abortion"  OR  "D&E"  OR  "dilation and evacuation"  OR  "mifepristone"  OR  "misoprostol"  OR  "terminat* N/3 pregnan*" ) )  AND  TITLE-ABS-KEY ( caesarean  OR  cesarean  OR  c-section )  AND  ( perforation  OR  rupture  OR  hysterectom*  OR  laparotom*  OR  bleed*  OR  "blood loss"  OR  haemorrhage  OR  hemorrhage  OR  transfus*  OR  dehiscence  OR  complication*  OR  death  OR  safety  OR  outcomes  OR  safe  OR  prevent*  OR  manag*  OR  ripen*  OR  dilator  OR  dilation  OR  adverse  OR  major  OR  serious  OR  sequelae  OR  "retained products"  OR  incomplete  OR  laceration  OR  accreta  OR  previa  OR  praevia ) )  AND NOT  INDEXTERMS ( animals  OR  animal )  AND  ( EXCLUDE ( DOCTYPE ,  "re" )  OR  EXCLUDE ( DOCTYPE ,  "le" )  OR  EXCLUDE ( DOCTYPE ,  "ed" )  OR  EXCLUDE ( DOCTYPE ,  "sh" )  OR  EXCLUDE ( DOCTYPE ,  "ch" )  OR  EXCLUDE ( DOCTYPE ,  "bk" ) )  AND  ( LIMIT-TO ( LANGUAGE ,  "English" ) )

*Searched on 22/07/2024

EMBASE search strategy for abortion outcomes and management after previous caesarean section*

| #1 | 'induced abortion'/exp/mj OR 'therapeutic abortion'/exp/mj OR 'pregnancy termination'/exp/mj |
| --- | --- |
| #2 | Induced abortion.mp OR therapeutic abortion.mp OR pregnancy termination.mp |
| #3 | #1 or #2 |
| #4 | 'cesarean section'/exp/mj |
| #5 | cesarean:ti,ab,kw OR caesarean:ti,ab,kw OR c-section:ti,ab,kw |
| #6 | #4 or #5 |
| #7 | (perforat* OR rupture OR hysterectom* OR laparotom* OR bleed* OR 'blood loss' OR haemorrhage OR hemorrhage OR transfus* OR dehiscence OR complication* OR death OR safety OR outcomes OR safe OR prevent* OR manag* OR ripen* OR dilator OR dilation OR adverse OR serious OR major OR sequelae OR 'retained products' OR incomplete OR laceration OR accreta OR previa OR praevia).ab |
| #8 | #3 AND #6 |
| #9 | #7 AND #8 |
| #10 | #7 and # AND [English]/lim AND [humans]/lim |

*Searched on 22/07/2024
