# Supplementary material for: Induced Abortion After Previous Caesarean Section: A Scoping Review
Source: Aust N Z J Obstet Gynaecol. 2025 Apr 11;65(5):564–85. doi: 10.1111/ajo.70013 (PMC12723096; doi:10.1111/ajo.70013)

**Appendix S2.** Risk of Bias and Quality Appraisal

Quality appraisal of case series included in the scoping review using the JBI critical appraisal tool[^200^](#_ENREF_212)

Risk of bias assessment of observational studies included in the scoping review using the ROBINS-E tool^201^ and robvis traffic light plot[^202^](#_ENREF_214)


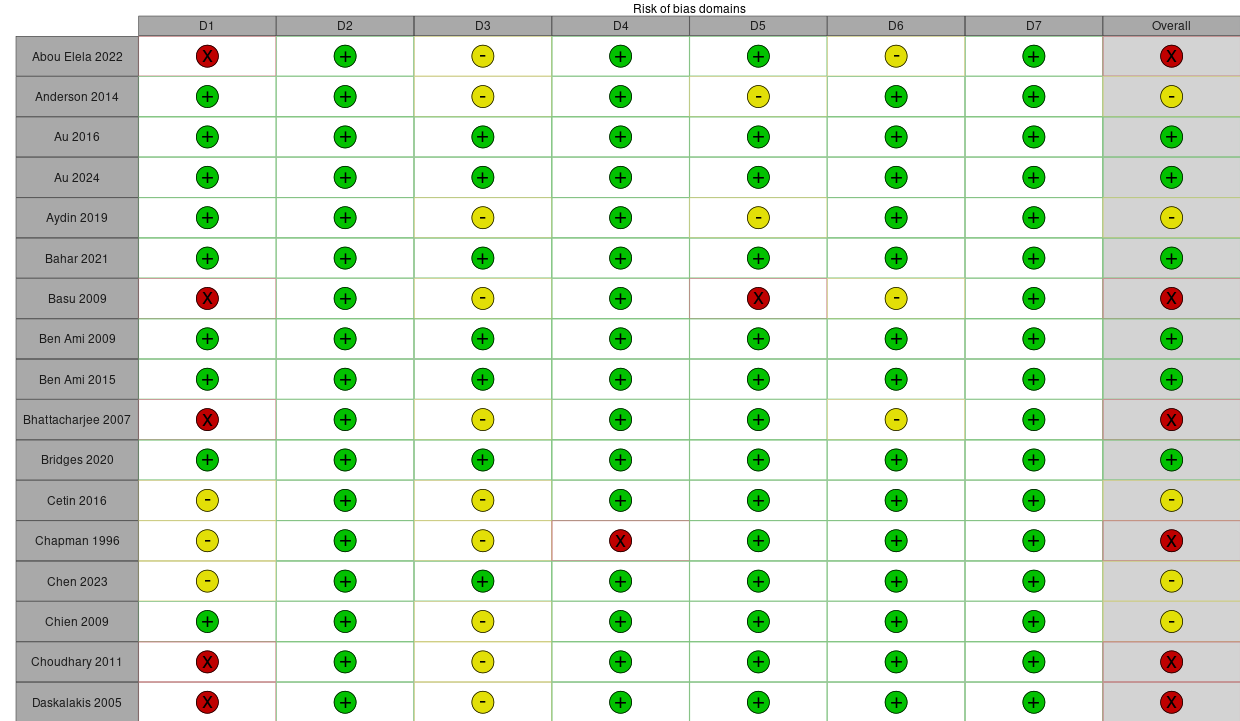


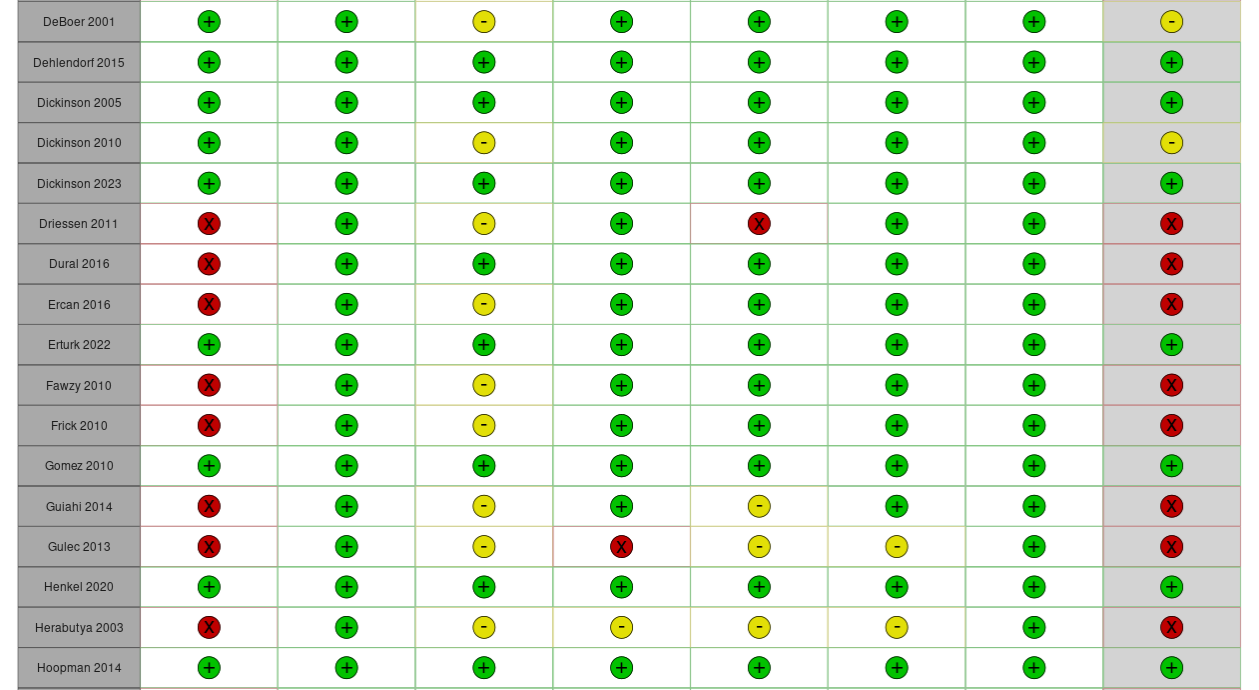

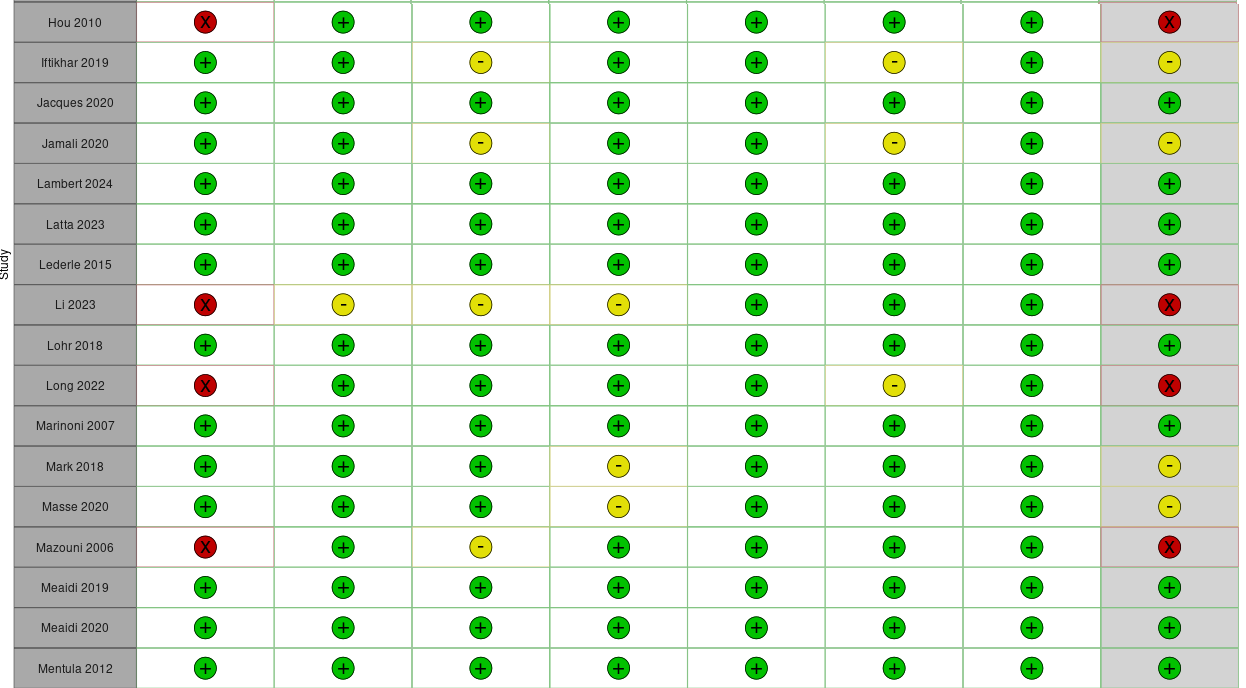

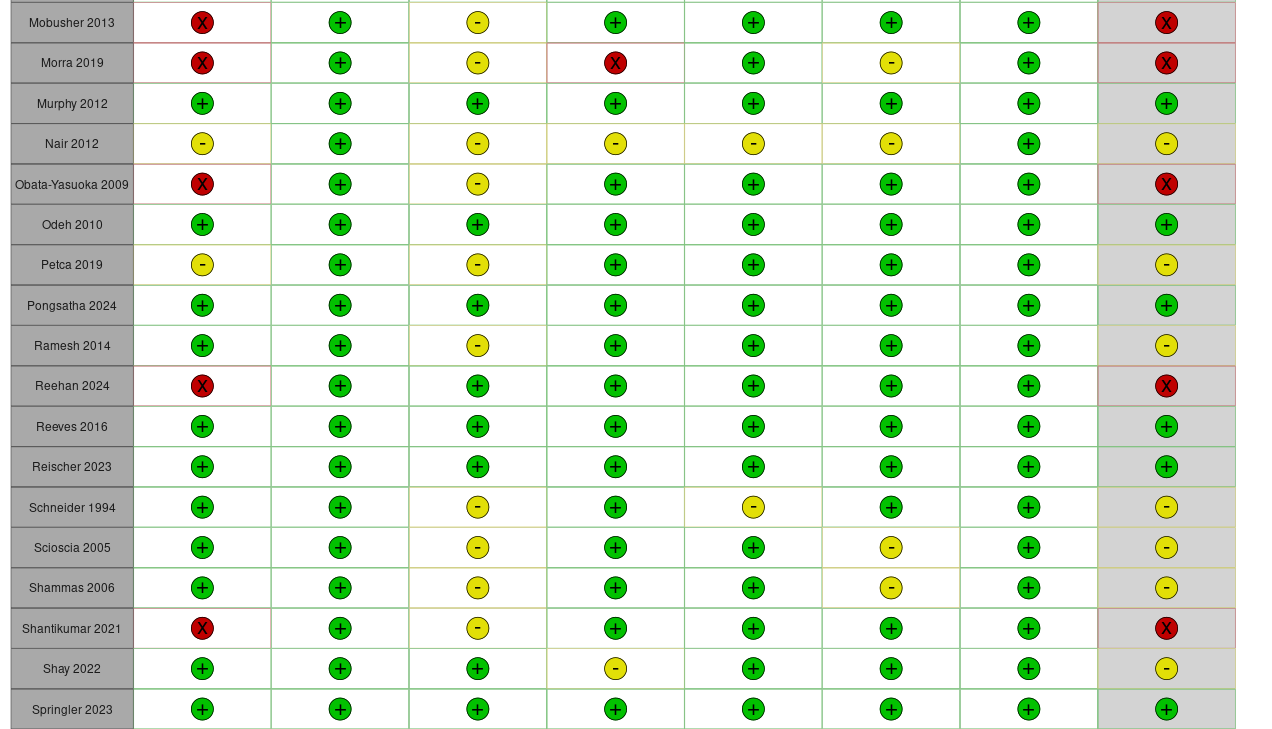

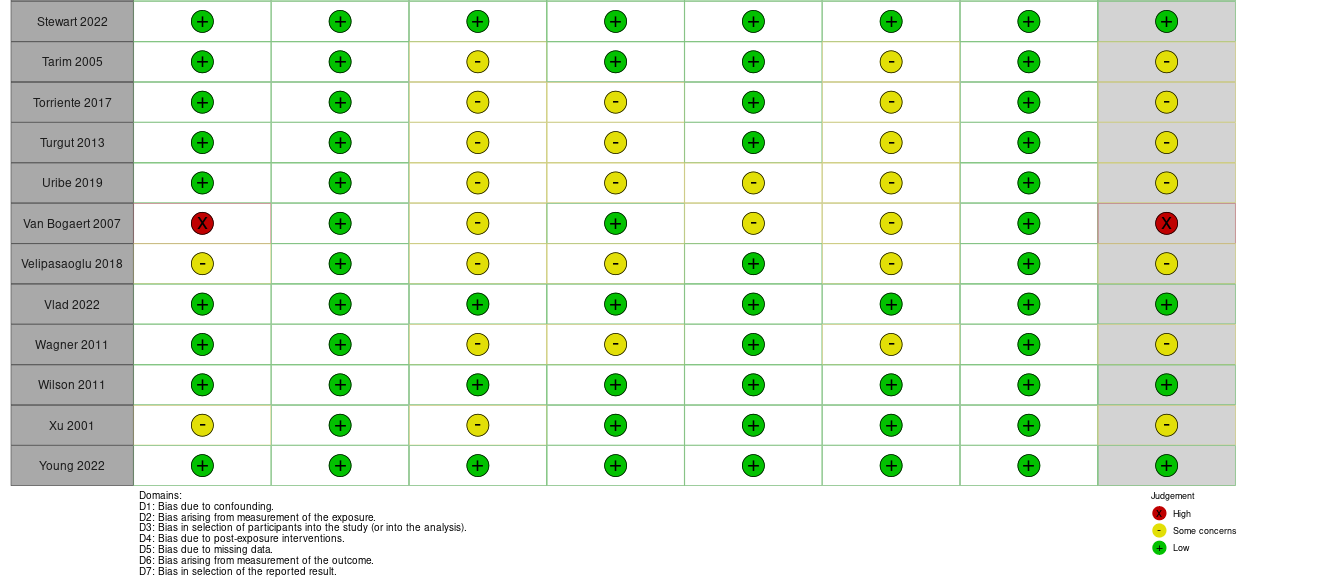


Risk of bias assessment for non-randomised interventional studies included in the scoping review using ROBINS-I tool[^19^](#_ENREF_19) and robvis traffic light plot[^202^](#_ENREF_214)


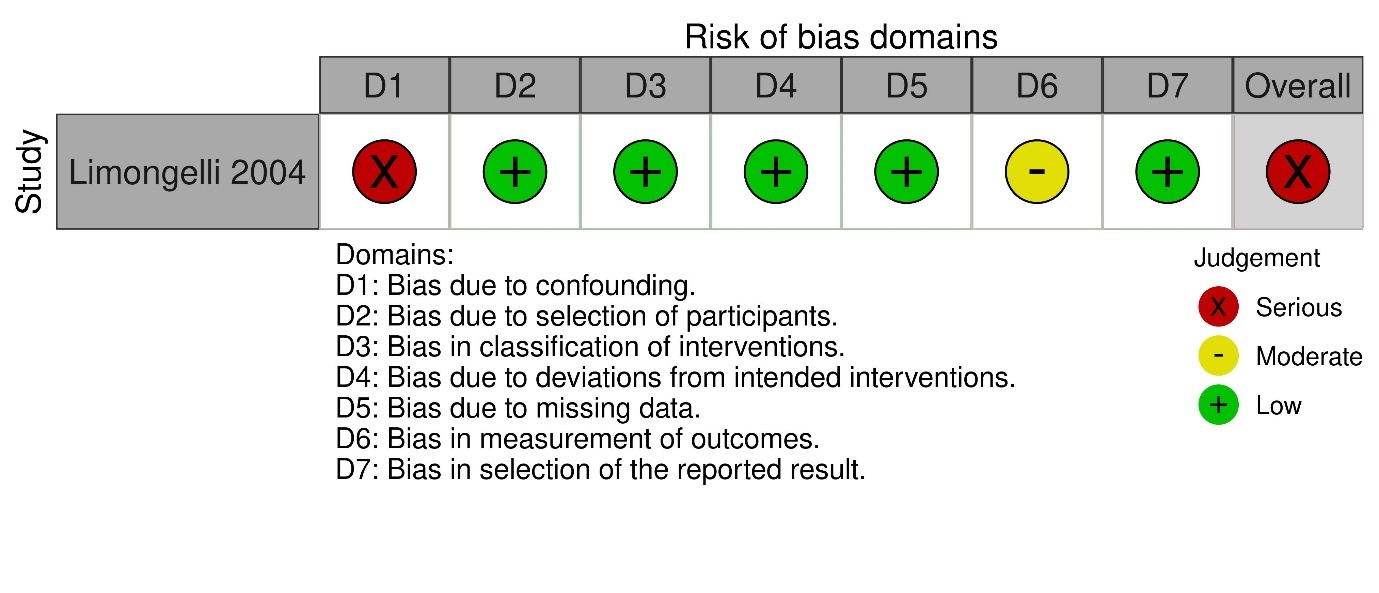


Risk of bias assessment for randomised controlled trials included in the scoping review using RoB 2.0 tool[^18^](#_ENREF_18) and robvis traffic light plot^202^


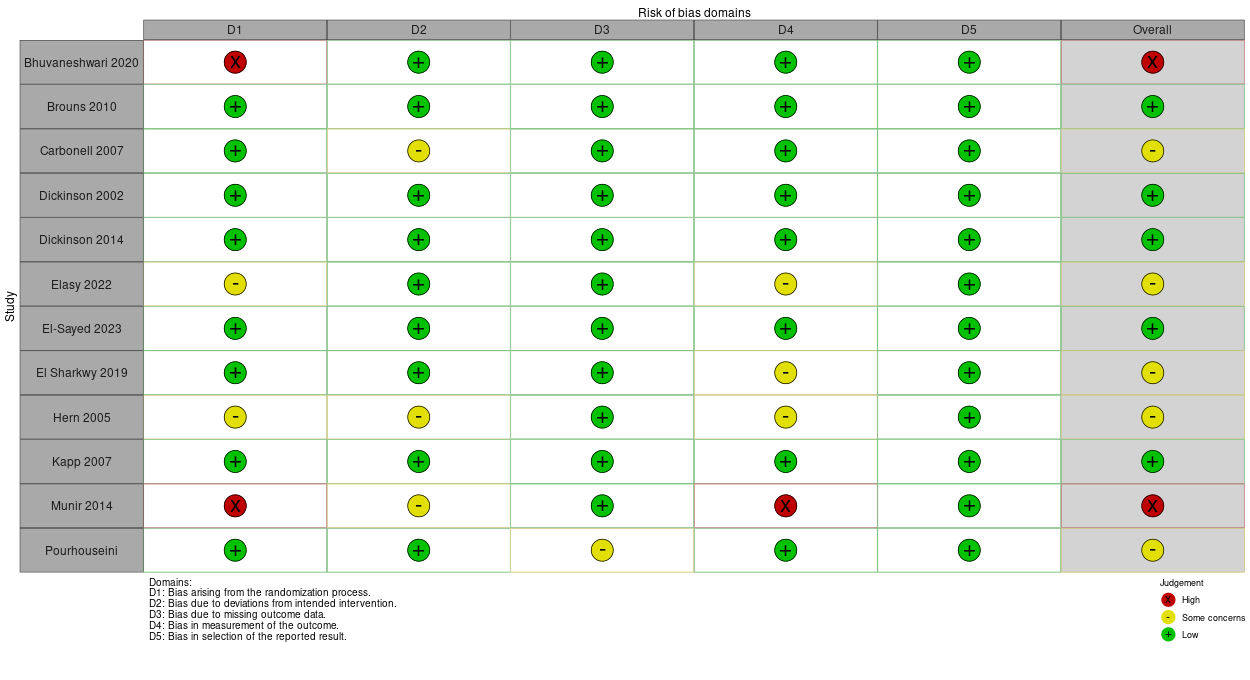

Supplement: Supplementary file 2 — Appendix S2 [file AJO-65-564-s007.docx]
