# Supplementary material for: Induced Abortion After Previous Caesarean Section: A Scoping Review
Source: Aust N Z J Obstet Gynaecol. 2025 Apr 11;65(5):564–85. doi: 10.1111/ajo.70013 (PMC12723096; doi:10.1111/ajo.70013)
Supplement: Supplementary file 3 — Figure S1 [file AJO-65-564-s001.docx]

**Figure S1**. Risk of need for surgical intervention during first or second trimester MToP in women with and without prior CS


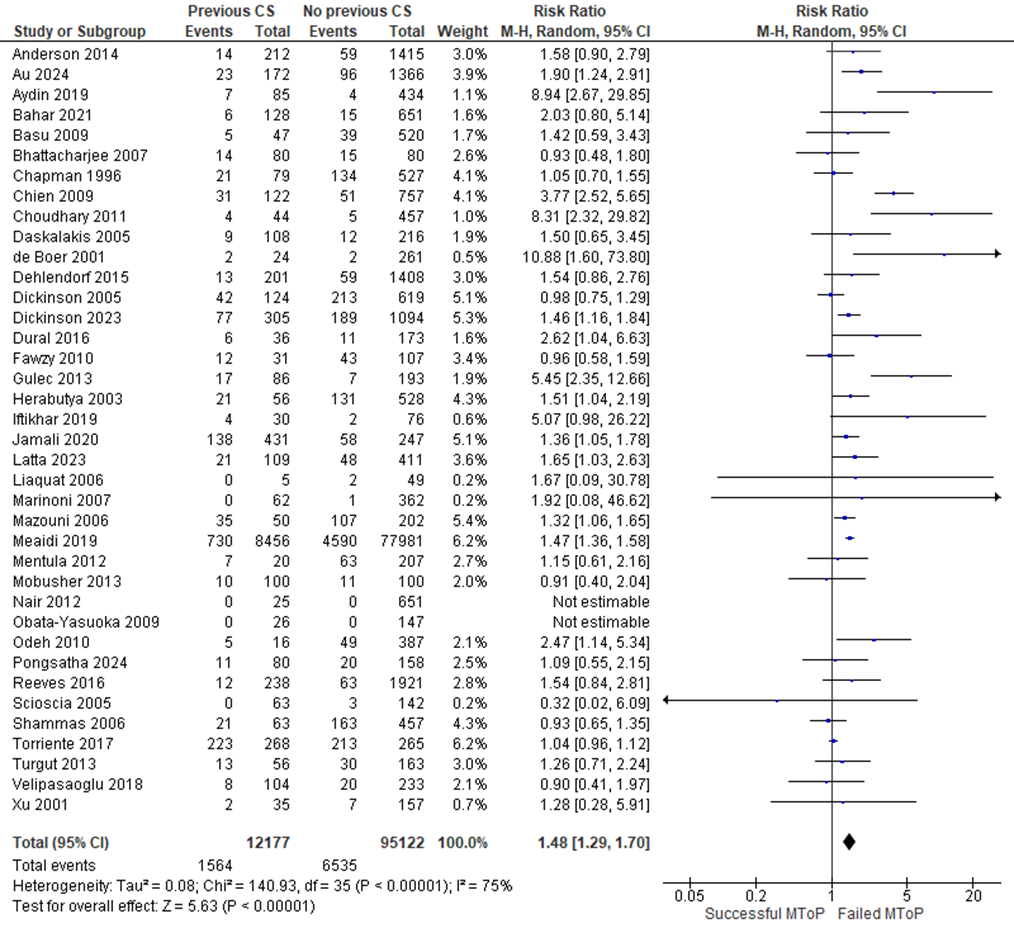


# 
