# Supplementary material for: Induced Abortion After Previous Caesarean Section: A Scoping Review
Source: Aust N Z J Obstet Gynaecol. 2025 Apr 11;65(5):564–85. doi: 10.1111/ajo.70013 (PMC12723096; doi:10.1111/ajo.70013)
Supplement: Supplementary file 5 — Table S2 [file AJO-65-564-s003.docx]

**Table S2.** Eligibility criteria

|  | **Inclusion criteria** | **Exclusion criteria** |
| --- | --- | --- |
| Population | Human studies that include women with at least one previous caesarean section | - Animal studies - Studies that do not include women with previous caesareans |
| Intervention/exposure | Medical or surgical termination of pregnancy up to 28 weeks gestation | - Studies related to management of known caesarean scar ectopic pregnancy* - Studies on management of miscarriage or intrauterine fetal death - Studies describing abortion methods other than prostaglandin induced abortion or surgical abortion techniques |
| Comparator | Controlled and uncontrolled studies will be included |  |
| Outcome | Complications: rates, prevention, management | Studies that do not include information relating to prevention, management or rates of adverse outcomes for women with previous caesareans |
| Timeframe | All reported timeframes will be included |  |
| Methodology | Primary sources of evidence including:   - Descriptive observational studies: case reports, case series, cross-sectional studies. - Analytical observational study designs (prospective or retrospective case-control, cohort, cross-sectional) or experimental study designs. | - Secondary sources of evidence including systematic reviews and meta-analyses - Opinions, letters to the editor, book chapters, news articles - Protocols and guidelines |
| Setting | Outpatient, hospital or day surgical settings will be included |  |
| Publication status | Studies will be included regardless of publication status |  |
| Time period | No date limits will apply |  |
| Language | Studies published in English | Studies published in languages other than English |

**The decision to exclude women undergoing treatment of known caesarean scar pregnancy (CSP) was made, as this would then include a large number of papers specifically regarding treatment of this particular condition, which is outside the scope of this review. However, it must be noted that some women with CSP may have initially presented seeking abortion, and subsequently diagnosed and treated for CSP, and hence will not be captured in this data. However, the study will include studies and case reports of women undergoing abortion with undiagnosed CSP that became apparent after the process of abortion had begun*.
