# Supplementary material for: Induced Abortion After Previous Caesarean Section: A Scoping Review
Source: Aust N Z J Obstet Gynaecol. 2025 Apr 11;65(5):564–85. doi: 10.1111/ajo.70013 (PMC12723096; doi:10.1111/ajo.70013)
Supplement: Supplementary file 6 — Table S3 [file AJO-65-564-s005.docx]

**Table S3.** Data extraction fields and variables

| **Data variables** | **Values** |
| --- | --- |
| Author, year, country of origin |  |
| Aims/purpose | Establish incidence of adverse outcomes, prevent or manage complications |
| Methodology | Case report, case series, cross-sectional, case-control, cohort, randomized, guideline/protocol |
| Sample size | Number of women included in the study with previous caesarean |
| Study population | Gestation range, parity, type and number of previous caesareans, presence of placenta praevia/accreta |
| Method of abortion | Medical or surgical  Medical – type and dose of prostaglandin  Surgical – cervical priming type, dose and duration. And method of abortion: dilation and curettage (D&C) or dilation and evacuation (D&E), hysterotomy, gravid hysterectomy. |
| Interventions to improve safety | If described. For example description of additional imaging, alteration of prostaglandin dose, use of osmotic dilators, intraoperative ultrasound, uterine artery embolisation. |
| Findings/outcomes | Incidence of complications: haemorrhage/blood loss, perforation, uterine rupture, retained products of conception, laparotomy, hysterectomy.  Management of complications |
| Relevant additional variables |  |
| Key findings and conclusions |  |
| Gaps in evidence |  |
