# Supplementary material for: Induced Abortion After Previous Caesarean Section: A Scoping Review
Source: Aust N Z J Obstet Gynaecol. 2025 Apr 11;65(5):564–85. doi: 10.1111/ajo.70013 (PMC12723096; doi:10.1111/ajo.70013)
Supplement: Supplementary file 7 — Table S4 [file AJO-65-564-s008.docx]

**Table S4.** Summary of case reports included in scoping review

| **Authors** | **Publication date** | **MToP/SToP** | **Method of abortion** | **Gestation** | **Parity** | **Number of prior CS** | **Type of prior CS** | **Abnormal placenta** | **Outcomes** |  |
| --- | --- | --- | --- | --- | --- | --- | --- | --- | --- | --- |
| Alsibiani, S[^197^](#_ENREF_197) | 2009 | MToP | Overnight foley catheter and syntocinon for ripening followed by 800mcg oral misoprostol | 21 weeks | 11 | 3 | Unspecified | No | Surgical evacuation required for active bleeding, unquantified |  |
| Amarosa et al[^163^](#_ENREF_163) | 2015 | MToP | Intracardiac potassium and 3 doses 50mg/m2 systemic (IM) MTX | 21 weeks | 2 | 2 | LSCS | Percreta | 4 months hospitalisation then developed VTE, cystotomy, and  chorioamnionitis requiring gravid hysterectomy |  |
| Anant et al (Case 1)[^154^](#_ENREF_154) | 2019 | MToP | Unknown | 6 weeks | 1 | 1 | Unspecified | Undiagnosed | Undiagnosed CSP and heavy bleeding at time of D&C for  suspected RPOC |  |
| Anant et al (Case 2)[^154^](#_ENREF_154) | 2019 | MToP | Unknown | 7 weeks | 1 | 1 | Unspecified | Undiagnosed | Undiagnosed CSP and heavy bleeding requiring D&C then  laparotomy. Blood transfusion 3 units. |  |
| Anant et al (Case 3)[^154^](#_ENREF_154) | 2019 | SToP | D&C | 8 weeks | 1 | 1 | Unspecified | Undiagnosed | Undiagnosed CSP diagnosed due to ongoing bleeding after  D&C managed successfully with systemic MTX |  |
| Ataei et al[^155^](#_ENREF_155) | 2018 | SToP | D&E | 15 weeks | 2 | 2 | Unspecified | Undiagnosed | Perforation and undiagnosed percreta |  |
| Balkanli-Kaplan et al[^156^](#_ENREF_156) | 2006 | SToP | D&C | 7 weeks | 1 | 1 | Unspecified | Undiagnosed | Undiagnosed percreta with persistent trophoblastic tissue  causing bleeding 8 months later |  |
| Bedaiwy at al[^164^](#_ENREF_164) | 2011 | SToP | Gravid hysterectomy | 17 weeks | 5 | 5 | 1 classical followed  by 4 uterine ruptures | Placenta overlying  previous classical scar with no sonographic evidence of accreta | Uncomplicated gravid hysterectomy, accreta confirmed on  histopathology |  |
| Berghahn et al[^22^](#_ENREF_22) | 2001 | SToP | D&E | 23 weeks | 2 | 2 | LSCS | No | Uterine rupture from cervical ripening, massive blood loss,  transfusion, infection |  |
| Bika et al (case 1)[^23^](#_ENREF_23) | 2014 | MToP | 200mg mifepristone followed  by 2 x doses 800mcg misoprostol vaginally | 10 weeks | 2 | 2 | LSCS | IUP low in fibroid  uterus | Failed MToP followed by SToP complicated by heavy  bleeding and dehiscence |  |
| Bika et al (case 2)[^23^](#_ENREF_23) | 2014 | MToP | 200mg mifepristone followed  by 4 x doses 200mcg misoprostol 6 hourly | 22 weeks | 1 | 1 | LSCS | No | Uterine rupture |  |
| Caruso et al[^24^](#_ENREF_24) | 2021 | MToP | 200mg mifepristone followed  by 3 x 400mcg misoprostol orally every 3 hours | 22 weeks | 1 | 1 | LSCS | No | Vesicouterine rupture with fetal parts in bladder |  |
| Chang et al[^136^](#_ENREF_136) | 2008 | SToP | D&C | 7 weeks | 2 | 2 | Unspecified | Undiagnosed | Perforation through CS scar and bowel damage |  |
| Chantraine et al (case 1)[^165^](#_ENREF_165) | 2012 | MToP | UAE, MTX and fundal hysterotomy | 17 weeks |  |  | Unspecified | Placenta percreta | Successful ToP without hysterectomy and placenta left in situ |  |
| Chantraine et al (case 2)[^165^](#_ENREF_165) | 2012 |  | UAE, MTX and fundal hysterotomy | 14 weeks |  |  | Unspecified | Placenta percreta | Successful ToP without hysterectomy and placenta left in situ;  blood transfusion |  |
| Chen et al[^25^](#_ENREF_25) | 1999 | MToP | 200mcg misoprostol vaginally  x 1 dose | 23 weeks | 2 | 2 | LSCS | No | Uterine rupture |  |
| Ciebiera et al[^26^](#_ENREF_26) | 2018 | MToP | 200mcg misoprostol vaginally  every 3 hours | 20 weeks | 1 | 1 | Unspecified | No | Uterine dehiscence |  |
| Daskalakis et al[^27^](#_ENREF_27) | 2005 | MToP | 400mcg oral misoprostol +  400mcg vaginal misoprostol followed by 400mcg vaginal misoprostol 8 hours later | 23 weeks | 1 | 1 | Unspecified | No | Uterine rupture |  |
| Einenkel et al[^157^](#_ENREF_157) | 2005 | SToP | D&C | 13 weeks | 2 | 2 | LSCS | Undiagnosed | Undiagnosed CSP and heavy bleeding at time of D&C |  |
| El-Matary et al[^28^](#_ENREF_28) | 2006 | MToP | 200mg mifepristone followed by  800mcg misoprostol PV and 5 x doses 400mcg misoprostol PV 4 hourly | 14 weeks | 1 | 1 | LSCS | No | Uterine rupture |  |
| Faraj et al[^39^](#_ENREF_39) | 2024 | MToP | 200mcg PO misoprostol | 10 weeks | 1 | 1 | Unspecified | No | Uterine rupture |  |
| Forster et al[^137^](#_ENREF_137) | 1989 | SToP | D&C | 12 weeks | 2 | 1 | Unspecified | No | Perforation through CS scar with extrusion of fetal parts  and abscess formation |  |
| Golshahi et al (case 1)[^29^](#_ENREF_29) | 2022 | MToP | 100mcg sublingual misoprostol followed by 4 x doses 200mcg 4 hourly | 21 weeks | 2 | 2 | LSCS | No | Uterine rupture |  |
| Golshahi et al (case 2)[^29^](#_ENREF_29) | 2022 | MToP | 4 x doses 200mcg sublingual  misoprostol | 23 weeks | 2 | 2 | LSCS | No | Uterine rupture |  |
| Golshahi et al (case 3)[^29^](#_ENREF_29) | 2022 | MToP | 3 x doses 400mcg sublingual  misoprostol every 5 hours | 20 weeks | 1 | 1 | LSCS | No | Uterine rupture |  |
| Gosakan et al[^30^](#_ENREF_30) | 2006 | MToP | 200mg mifepristone followed by  800mcg PV misoprostol then 4 x doses 400mcg oral misoprostol 3 hourly, then 3 hourly gemeprost 1mg | 19 weeks | 2 | 2 | LSCS | No | Uterine rupture |  |
| Hanstede et al[^158^](#_ENREF_158) | 2008 | SToP | D&E | 18 weeks | 2 | 2 | Unspecified | No | Undiagnosed cervical pregnancy |  |
| Jiang et al[^31^](#_ENREF_31) | 2015 | MToP | 300mg mifepristone in divided doses  followed by 6 x doses 200mcg PO misoprostol hourly | 16 weeks | 1 | 1 | LSCS | Suspected possible accreta | Uterine rupture |  |
| Jwarah et al[^32^](#_ENREF_32) | 2000 | SToP | 800mcg vaginal misoprostol for  cervical priming prior to D&C | 8 weeks | 2 | 1 | Unspecified | No | Uterine rupture involving both uterine arteries |  |
| Kaba et al[^161^](#_ENREF_161) | 2023 | MToP | Not stated | “Second-trimester” | 4 | 4 | Unspecified | Yes | Undiagnosed accreta requiring laparotomy and hysterectomy |  |
| Kerr et al[^166^](#_ENREF_166) | 1996 | SToP | D&E | 18 weeks | 4 | 4 | Unspecified | Placenta praevia and suspected accreta | Severe haemorrhage requiring subtotal hysterectomy  and embolisation; cystotomy; massive blood transfusion |  |
| Lichtenberg et al (case 1)[^33^](#_ENREF_33) | 2004 | SToP | Laminaria followed by D&E | 21 weeks | 1 | 1 | LSCS | No | Uterine dehiscence |  |
| Lichtenberg et al (case 2)[^33^](#_ENREF_33) | 2004 | SToP | Laminaria followed by D&E | 20 weeks | 3 | 3 | Unspecified | Anterior placenta praevia | Uterine rupture; sepsis; pulmonary oedema/ARDS |  |
| Limbachiya et al[^40^](#_ENREF_40) | 2023 | MToP | 200mg mifepristone followed by 600mcg PV misoprostol | 18 weeks | 1 | 1 | Unspecified | No | Vesicouterine rupture |  |
| Matsuzaki et al[^167^](#_ENREF_167) | 2014 | MToP | Gemeprost | 20 weeks | 1 | 1 | Unspecified | Placenta praevia and suspected accreta | Massive haemorrhage secondary to accreta |  |
| Nayki et al[^34^](#_ENREF_34) | 2004 | MToP | 4 x doses 200mcg vaginal misoprostol | 26 weeks | 1 | 1 | LSCS | No | Uterine rupture |  |
| Orellana et al[^159^](#_ENREF_159) | 2020 | SToP | D&C | 7 weeks | 2 | 2 | Unspecified | Undiagnosed | Undiagnosed CSP diagnosed due to ongoing bleeding  after D&C |  |
| Oteri et al[^198^](#_ENREF_198) | 1999 | MToP | 600mg mifepristone followed by  3 x 400mcg PO misoprostol 4 hourly | 15 weeks | 5 | 2 | Unspecified | No | Successful ToP |  |
| Poudel et al[^35^](#_ENREF_35) | 2020 | MToP | 200mg mifepristone followed  by 23 x doses 400mcg PO misoprostol 3 hourly, followed by 2 further attempts at induction in sibsequent admission | 16 weeks | 2 | 2 | Unspecified | Undiagnosed | Uterine rupture and undiagnosed accreta |  |
| Rajesh et al[^36^](#_ENREF_36) | 2002 | MToP | Mifepristone followed by gemeprost;  no dose given | 20 weeks | 3 | 3 | LSCS | No | Uterine rupture |  |
| Seto et al[^189^](#_ENREF_189) | 2013 | MToP | Dilapan followed by PV misoprostol  50mcg, 100mcg, 150mcg at 0, 4, 8 hours | 14 weeks | 2 | 1 | Classical | No | Successful ToP |  |
| Sharpless et al[^162^](#_ENREF_162) | 2022 | SToP | 400mcg buccal misoprostol followed by D&C | 12 weeks | 4 | 4 | Unspecified | No | AVM over prior CS scar diagnosed after haemorrhage from SToP; managed with uterine artery embolisation |  |
| Shojai et al[^160^](#_ENREF_160) | 2012 | SToP | D&C following failed MToP | 8 weeks | 2 | 2 | Unspecified | Undiagnosed | Undiagnosed placenta percreta and massive haemorrhage |  |
| Stitely et al[^37^](#_ENREF_37) | 2015 | SToP | D&E with 400mcg PO misoprostol  priming | 13 weeks | 1 | 1 | LSCS | No | Uterine rupture and haemorrhage |  |
| Tocce et al [^168^](#_ENREF_168) | 2009 | Gravid hysterectomy | Gravid hysterectomy | 17 weeks | 1 | 1 | Unspecified | Placenta increta | Successful gravid hysterectomy; minor wound infection |  |
| Zohav et al[^38^](#_ENREF_38) | 2016 | MToP | 800mg PV misoprostol followed  by 3 x 400mcg SL misoprostol 3 hourly | 13 weeks | 1 | 1 | LSCS | No | Uterine rupture |  |

*MToP, medical termination of pregnancy; SToP, surgical termination of pregnancy; CS, caesarean section; IM, intramuscular; MTX, methotrexate; LSCS, lower segment caesarean section; VTE, venous thromboembolus; CSP, caesarean scar pregnancy; D&C, dilation and curettage; (R)POC, (retained) products of conception; UAE, uterine artery embolisation; ToP, termination of pregnancy; D&E, dilation and evacuation; ARDS, acute respiratory distress syndrome; PO, per oral; PV, per vaginal; SL, sublingual; IUP, intrauterine pregnancy.*
