# Supplementary material for: Induced Abortion After Previous Caesarean Section: A Scoping Review
Source: Aust N Z J Obstet Gynaecol. 2025 Apr 11;65(5):564–85. doi: 10.1111/ajo.70013 (PMC12723096; doi:10.1111/ajo.70013)
Supplement: Supplementary file 8 — Table S5 [file AJO-65-564-s006.docx]

**Table S5.** **Table S5.** Summary of included original research articles

| **Authors** | **Country** | **Date** | **Study design** | **Number of participants with prior CS** | **Number of participants without prior CS** | **MToP/SToP** | **Gestation** | **Main findings** |  |
| --- | --- | --- | --- | --- | --- | --- | --- | --- | --- |
| Abou Elela et al[^87^](#_ENREF_87) | Egypt | 2022 | Prospective cohort | 50 | 50 | MToP | 13-26 weeks | Previous CS was associated with increased abortion time compared to people without previous CS. No cases of rupture. |  |
| Anderson et al[^129^](#_ENREF_129) | USA | 2014 | Retrospective cohort | 211^†^ | 1416 | MToP | First-trimester^‡^ | No significant association with uterine scarring  and need for additional intervention (OR 1.7, CI 0.87-3.2) |  |
| Au et al[^199^](#_ENREF_199) | Taiwan | 2016 | Retrospective cohort | 183 | No | MToP | 6-8 weeks | CSD was associated with increased risked of  failed MToP (OR 3.32), with 57% failure rate if defect in myometrial thickness ratio <30%. Number of caesareans not related to risk of failure. |  |
| Au et al[^47^](#_ENREF_47) | Taiwan | 2024 | Retrospective cohort | 140 | 940 | MToP | <13 weeks | Previous CS was associated with significant increase in risk of retained products/need for surgical intervention after early MToP. No cases of rupture. |  |
| Aydin et al[^48^](#_ENREF_48) | Turkey | 2019 | Retrospective cohort | 85 | 434 | MToP | 19.19+/- 2.63 weeks^‡^ | Success rates of ToP were lower in women with previous CS (p=<0.001) and risk of haemorrhage requiring transfusion higher (p=0.03). Overall safe and >90% effective for women with previous CS. |  |
| Bahar et al[^49^](#_ENREF_49) | Israel | 2021 | Retrospective cohort | 128 | 651 | MToP | 13-26 weeks | Women with previous CS more likely to have failed MToP (p=0.03). Previous CS associated with increased risk bleeding without need for transfusion (p=0.05) but not cervical laceration, retained placenta, blood transfusion or infection. One case of undiagnosed accreta. |  |
| Basu et al[^50^](#_ENREF_50) | South Africa | 2009 | Cohort | 47 | 520 | MToP | 16 +/- 2 weeks | Successful abortion during first admission in  89% of women with previous CS, no difference between women with and without previous CS |  |
| Ben-Ami et al[^138^](#_ENREF_138) | Israel | 2009 | Retrospective cohort | 91 | 545 | SToP | 17-24 weeks | Operative time, number of laminaria tents and operative complications did not differ between the groups with and without previous CS. No major complications in any of the groups. |  |
| Ben-Ami et al[^186^](#_ENREF_186) | Israel | 2015 | Case-control | 27 | 213 | SToP | 14-24 weeks | Previous CS was a significant risk factor for inadequate dilation after laminaria placement OR 6.4 (p=<0.001), nulliparity also a risk factor |  |
| Berghella et al[^8^](#_ENREF_8) | USA | 2009 | Case series | 17 | No | MToP | 16-28 weeks | No uterine ruptures |  |
| Bhattacharjee et al[^51^](#_ENREF_51) | India | 2007 | Prospective cohort | 80 | 80 | MToP | 13-26 weeks | No significant differences between the groups  with and without previous CS: blood loss/transfusion, sepsis, failed abortion, need for operative management |  |
| Bhuvaneswari et al[^52^](#_ENREF_52) | India | 2020 | Randomised comparative study | 100 | No | MToP | 13-26 weeks | No significant difference in side effects,  failed abortion or complication rates between those with Foley catheter vs misoprostol; Mean abortion time shorter with mifepristone and misoprostol compared with Foley catheter + misoprostol (9.09 hours vs 11 hours p<0.01) Rupture rate 1% (occurred in woman in mifepristone group) |  |
| Boulot et al[^119^](#_ENREF_119) | France | 1993 | Cohort | 23 | No | MToP | "mid and third trimesters"^‡^ | 87% achieved vaginal birth, one case of rupture and one case of haemorrhage |  |
| Bridges et al[^200^](#_ENREF_200) | USA | 2020 | Retrospective cohort | 31 | 130 | SToP | 14-24 weeks | Previous CS not associated with significant  increase in EBL; each week of gestation associated with significant increase in EBL |  |
| Brouns et al[^88^](#_ENREF_88) | Netherlands | 2010 | Randomised controlled trial | 12 | 164 | MToP | 14-24 weeks | Trial compared mifepristone followed by 200mcg vs 400mcg misoprostol. No ruptures occurred. Abortion time longer with lower dose |  |
| Carbonell et al[^201^](#_ENREF_201) | Spain | 2007 | Randomised controlled trial | 60 | 840 | SToP | 12-20 weeks | None of the women with previous CS experienced complications of D&E. |  |
| Cayrac et al[^120^](#_ENREF_120) | France | 2011 | Cohort | 67 | No | MToP | 14-37 weeks | 95.5% of women delivered vaginally; rupture rate 4.8% (3 women); transfusion rate 3% |  |
| Cetin et al[^53^](#_ENREF_53) | Turkey | 2016 | Case-control | 77 | 186 | MToP | 12-24 weeks | One case of rupture in the higher dose group,  this patient had 3 previous CS; No difference in failure rates between the two regimens |  |
| Chapman et al[^115^](#_ENREF_115) | USA | 1996 | Retrospective cohort | 79 | 527 | MToP | 11-28 weeks | Previous CS was a risk factor for blood transfusion OR 2.3 (p=0.04) but not associated with increased risk surgical intervention compared with women without CS; rupture rate 3.8% of women with previous CS |  |
| Chen et al[^89^](#_ENREF_89) | Taiwan | 2023 | Retrospective cohort | 204 | No | MToP | 14-27 weeks | No significant differences in successful abortion between groups using the addition of a cervical ripening catheter compared to mifepristone-misoprostol regimens. 1 case of rupture in a woman with prior CS who receiving mifepristone-misoprostol regimen. |  |
| Chien et al[^41^](#_ENREF_41) | Taiwan | 2009 | Retrospective cohort | 122 | 757 | MToP | 8 weeks or less | Previous CS was associated with increased  risk of failed early MToP compared to vaginally parous women (OR 3.33) and nulliparous women (OR 9.59) |  |
| Chodankar et al[^139^](#_ENREF_139) | Multiple | 2018 | Prospective observational | 80 | 403 | Both^§^ | 6-24 weeks | Dilapan-S use results in reduced dose of  misoprostol required for MToP and is also safe and effective as a cervical priming method alone prior to SToP. No serious adverse outcomes including in women with previous CS |  |
| Choudhary et al[^54^](#_ENREF_54) | India | 2011 | Retrospective cohort | 44 | 456 | MToP | 12-20 weeks | Overall complication rates were comparable  between women with and without uterine scar (p=0.76), however women without uterine scar had higher MToP success rates (p=0.00), 1 out of 44 women with previous CS experienced rupture (2%) |  |
| Cui et al[^169^](#_ENREF_169) | China | 2018 | Case series | 7^†^ | No | SToP | 13-28 weeks | Preoperative UAE used in 6 patients and all  received internal iliac ligation with 4 patients having intrauterine balloon tamponade. All patients experienced massive haemorrhage (average 2474ml) and 3 developed DIC. 3/7 patients required subtotal hysterectomy to control haemorrhage. |  |
| Daponte et al[^55^](#_ENREF_55) | South Africa | 2006 | Cohort | 85 | No | MToP | 14-20 weeks | All patients had successful MToP and no uterine  ruptures; no significant differences in abortion time between the higher and the lower dose groups |  |
| Daponte et al[^56^](#_ENREF_56) | South Africa | 2007 | Cohort | 21 | No | MToP | First and second trimester^‡^ | No cases of rupture or significant haemorrhage;  no significant differences in abortion time between women with one or multiple previous CS; complete abortion only 33.3% in first trimester suggesting this dose is not high enough for first trimester MToP with scarred uterus |  |
| Daskalakis et al[^57^](#_ENREF_57) | Greece | 2005 | Retrospective cohort | 108 | 216 | MToP | 17-24 weeks | No significant differences between the groups with and without previous CS: blood loss/transfusion, sepsis, failed abortion, need for operative management. 99.4% rate of vaginal delivery |  |
| Davey et al[^58^](#_ENREF_58) | UK | 1997 | Cohort | 22 | 245 | MToP | 12-24 weeks | One case of uterine rupture amongst the  women with previous CS |  |
| DeBoer et al[^122^](#_ENREF_122) | Netherlands | 2001 | Retrospective cohort | 23^¶^ | 261 | MToP | 14-43 weeks | One case of uterine rupture amongst the  women with previous CS; Previous CS associated with reduced success of MToP (p<0.001) but not blood loss or retained placenta |  |
| Dehlendorf et al[^130^](#_ENREF_130) | USA | 2015 | Retrospective cohort | 201 | 1408 | MToP | <9 weeks | Previous CS was not associated with increased risk of failure OR 1.79 (CI 0.83-3.87) |  |
| Dickinson et al[^86^](#_ENREF_86) | Australia | 2002 | Randomised controlled trial | 12 | 138 | MToP | 14-30 weeks | Previous CS did not significantly affect abortion time; no cases of rupture; one undiagnosed accreta in a woman with 4 x previous CS; 400mcg misoprostol 6 hourly lower abortion time than 200mcg doses |  |
| Dickinson et al[^59^](#_ENREF_59) | Australia | 2005 | Retrospective cohort | 124 | 619 | MToP | 14-28 weeks | No cases of rupture or need for hysterectomy; no significant differences in abortion time, haemorrhage, retained placenta, EBL or blood transfusion between women with and without previous CS |  |
| Dickinson et al[^60^](#_ENREF_60) | Australia | 2010 | Retrospective cohort | 81 | 307 | MToP | 14-28 weeks | No cases of rupture or hysterectomy |  |
| Dickinson et al[^202^](#_ENREF_202) | Australia | 2014 | Randomised controlled trial | 66 | 236 | MToP | 14-24 weeks | Oral misoprostol is associated with increase abortion time compared to sublingual and vaginal administration. Caesarean section was significantly associated with increased placental retention (P=0001) and longer abortion time. 1 case of rupture – this case is included in the Dickinson 2023 paper and not shown separately in tables analysing rupture rates. |  |
| Dickinson et al[^114^](#_ENREF_114) | Australia | 2023 | Retrospective cohort | 304 | 1095 | MToP | 13-28 weeks | Complications were more frequent in women with prior CS, including blood loss >1000ml OR 2.11 (CI 1.23-3.62) and retained placenta OR 1.44 (CI 1.05-1.99). Uterine rupture occurred in 4/304 women with prior CS (1.3%) |  |
| Domrose et al[^61^](#_ENREF_61) | Germany | 2012 | Cohort | 111 | No | MToP | 14-34 weeks | 90.1% successful MToP within 48 hours;  1 rupture; severe complication including haemorrhage 8.1% |  |
| Driessen et al[^121^](#_ENREF_121) | France | 2011 | Retrospective cohort | 112 | 1362 | MToP | 2nd and 3rd trimester^‡^ | Rupture rate 2.7%; 7-fold increase in major  complications in women with previous CS compared to those without |  |
| Dural et al[^117^](#_ENREF_117) | Turkey | 2016 | Retrospective cohort | 36 | 173 | MToP | 14-32 weeks | Women with previous CS more likely to need  additional measures to complete abortion (16.6% vs 6.3% P=0.05) but received lower doses of misoprostol than women without previous CS; 1 rupture in each group; no differences in success rates or abortion interval; no significant severe complications |  |
| Elasy et al[^91^](#_ENREF_91) | Egypt | 2022 | Randomised controlled | 158 | No | MToP | 14-24 weeks | Misoprostol vs cervical ripening catheter + oxytocin was associated with shorter abortion time. Complication rates were not significantly different between the two groups, with the exception of diarrhoea being more frequent in the misoprostol group. 3 reports of rupture among 79 women with prior CS receiving misoprostol. |  |
| El-Sayed et al[^92^](#_ENREF_92) | Egypt | 2023 | Randomised comparative study | 56 | No | MToP | 13-18 weeks | No significant differences in complications or complete abortion between those with Dilapan-oxytocin vs misoprostol regimens. No cases of rupture reported. |  |
| El Sharkwy et al[^62^](#_ENREF_62) | Egypt | 2019 | Randomised controlled trial | 78 | No | MToP | 20-27 weeks | Women in the combined group had lower  rates of incomplete abortion (p=0.03), shorter abortion time (p=0.01) requiring fewer doses of misoprostol (p=0.04), with no difference in side effects or complications |  |
| Ercan et al[^63^](#_ENREF_63) | Turkey | 2016 | Retrospective cohort | 144 | 234 | MToP | 14-24 weeks | Women in the combined group had shorter  abortion interval (p=0.03) and required less doses of misoprostol (p=<0.001); no difference in complications and no ruptures |  |
| Erturk et al[^93^](#_ENREF_93) | Turkey | 2022 | Retrospective cohort | 137 | 80 | MToP | 14-24 weeks | Successful MToP and complication rates not significantly different between women with and without prior CS using misoprostol alone. 1 rupture in a woman with >1 prior CS. |  |
| Esteve et al[^116^](#_ENREF_116) | Spain | 2008 | Cohort | 17 | 411 | MToP | 19-26 weeks | One woman with previous CS experienced  rupture |  |
| Fawzy et al[^64^](#_ENREF_64) | Egypt | 2010 | Retrospective cohort | 31 | 107 | MToP | 13-26 weeks | No difference between dose needed and  abortion time between women with and without previous CS; 3 women in the study group required hysterotomy 9.7% versus 0 in control group (p=0.03); no difference in haemorrhage or retained placenta |  |
| Frick et al[^146^](#_ENREF_146) | USA | 2010 | Prospective cohort | 643 | 2330 | SToP | 14-27 weeks | Major complications (transfusion, DIC, reoperation) 1.3%, one previous caesarean did not have a significant effect on risk of major complication but history of ≥2 previous CS 7-fold increase in odds of major complication (OR 7.4, CI 3.0-17.6) |  |
| Gao et al[^42^](#_ENREF_42) | China | 1999 | Cohort | 213 | No | MToP | 4-7 weeks | Complete abortion in 92.5%, incomplete  abortion 4.7% and failure 2.8%, similar to rates published in general population. No significant differences between groups with shorter or longer interval since CS. |  |
| Garofalo et al[^65^](#_ENREF_65) | Italy | 2018 | Retrospective cohort | 141 | 703 | Both | <22 weeks | Uterine rupture in 5.5% of women with  previous CS, significantly more common in women undergoing 2nd trimester ToP compared with early ToP |  |
| Gautam et al[^43^](#_ENREF_43) | India | 2003 | Cohort | 66 | No | MToP | <9 weeks | Successful abortion in 93.9%, severe bleeding  3.1%, no cases of uterine rupture |  |
| Gomez et al[^109^](#_ENREF_109) | Spain | 2010 | Retrospective cohort | 28 | 242 | MToP | 12-23 weeks | Increasing gestational age was associated with increased abortion time. No cases of rupture amongst those with prior CS |  |
| Guiahi et al[^147^](#_ENREF_147) | USA | 2014 | Retrospective cohort | 363 | 1597 | SToP | First trimester^‡^ | History of caesarean was a significant predictor for complication (resuscitation, perforation, EBL >100cc or cervical laceration) OR 1.9, CI 1.1-3.4 |  |
| Gulec et al[^66^](#_ENREF_66) | Turkey | 2013 | Retrospective cohort | 86 | 193 | MToP | 14-26 weeks | Success rate 96.4% in women with no previous CS, 81.7% if one previous CS and 76.9% if two previous CS. 3.5% of women with previous CS had rupture, all of whom had ≥2 previous CS. Longer abortion time if previous CS |  |
| Henkel[^203^](#_ENREF_203) | USA | 2020 | Retrospective cohort study | 5 | 84 | MToP | 15-27 weeks | Shorter mifepristone-misoprostol intervals significantly decrease abortion time without affecting induction time. No ruptures amongst those with prior CS. |  |
| Herabutya et al[^67^](#_ENREF_67) | Thailand | 2003 | Prospective cohort | 56 | 528 | MToP | 14-26 weeks | No difference in dose needed or abortion time between women with and without previous CS. Incomplete abortion higher in those with previous CS (37.5% vs 24.8% p<0.01). No cases of rupture. |  |
| Hern et al[^140^](#_ENREF_140) | USA | 2005 | Non-blinded controlled clinical trial | 76 | 6964 | SToP | 18-38 weeks | Misoprostol reduced blood loss and procedure  time with no cases of rupture or major complications. Previous CS increased risk of EBL >500ml p<0.0001 |  |
| Hoopman et al[^125^](#_ENREF_125) | Germany | 2014 | Retrospective cohort | 49 | 284 | MToP | Second and third trimester^‡^ | Previous CS associated with significant increase in abortion time (OR 11.7, CI 4.5-19.0) |  |
| Hou et al[^110^](#_ENREF_110) | China | 2010 | Prospective cohort | 5 | 95 | MToP | 13-16 weeks | 2-day mifepristone-misoprostol regimen resulted in less incomplete abortions than 1-day interval. No cases of rupture. |  |
| Hu et al[^170^](#_ENREF_170) | China | 2021 | Cohort | 51 | No | Both | 13-23 weeks | 31 women had MToP and 20 had planned  hysterotomy. 30/31 in MToP group avoided hysterotomy but 20/30 required D&E due to adherent placental tissue. No significant difference in EBL, transfusion rates, length of stay, HCG recovery time, need for hysterectomy. 7.8% required hysterectomy. |  |
| Iftikhar et al[^124^](#_ENREF_124) | Pakistan | 2019 | Prospective cohort | 30 | 76 | MToP | 13-26 weeks | Complication rates including blood loss >1000ml (p=0.001) and need for surgical intervention (p=0.05) were higher in the group with previous CS |  |
| Jacques et al[^95^](#_ENREF_95) | USA | 2020 | Retospective cohort | 13 | 68 | MToP | 14-24 weeks | No cases of rupture |  |
| Jamali et al[^68^](#_ENREF_68) | Iran | 2020 | Retrospective cohort | 431 | 247 | MToP | 14-24 weeks | EBL >500ml higher in women with ≥2 previous CS (OR 2.24, CI 1.11-4), also higher risk need for additional treatment to complete abortion (OR 3.3, CI 1.23-9.1), non-significant increase in rupture |  |
| Kapp et al[^111^](#_ENREF_111) | USA | 2007 | Randomised controlled trial | 3 | 29 | MToP | 18-23 weeks | Addition of mifepristone for second-trimester abortions reduces abortion time. No cases of rupture. |  |
| Kiley et al[^127^](#_ENREF_127) | USA | 2022 | Case series | 3 | 12 | MToP | 23-26 weeks | No cases of rupture. |  |
| Koh et al[^69^](#_ENREF_69) | Singapore | 2018 | Cohort | 339 | No | MToP | 14-23 weeks | Rupture rate 0.2% |  |
| Lambert et al[^145^](#_ENREF_145) | USA | 2024 | Retrospective cohort | 66 | 598 | SToP | 18-22 weeks | No clinically significant difference in procedure time or complications between adjuvant mifepristone or misoprostol with D&E after osmotic dilator placement the day prior. No cases of rupture. 1 perforation in this cohort (but not stated whether patient had prior CS). No direct comparison between women with and without previous CS for other outcomes |  |
| Latta et al[^97^](#_ENREF_97) | USA | 2023 | Retrospective cohort | 109 | 411 | MToP | 14-24 weeks | Higher rates of rupture among women with ≥2 prior CS (P=0.004). This study also included 11 patients with prior vertical uterine incision with no ruptures in this group. Trend in increased haemorrhage and retained placenta with increasing number of previous CS but not significant. |  |
| LeRoux et al[^123^](#_ENREF_123) | UK | 2001 | Retrospective cohort | 14 | 54 | MToP | 14-35 weeks | 3 women in the study experienced uterine  rupture, all of whom had previous CS |  |
| Lederle et al[^148^](#_ENREF_204) | USA | 2015 | Retrospective cohort | 978 | 3542 | SToP | 14-24 weeks | Major complications are uncommon at D&E,  and while obesity was not shown to be associated with increased risk of major complications, previous CS was risk factor for same (OR 1.8, CI 1.1-3.1). Additionally, 2 patients with multiple previous CS required hysterectomy for major complications. |  |
| Li et al[^176^](#_ENREF_176) | China | 2023 | Retrospective cohort | Not stated. 48 women with placenta accreta spectrum included in study – managed with either MToP or caesarean section |  | Caesarean (hysterotomy) (28) or MToP (20) +/- UAE | 13-28 weeks | No significant differences were observed in success rates between the transvaginal termination and caesarean section groups (80.00% vs. 92.86%, *P* = 0.38). Furthermore, no statistically significant differences were observed in the success rates (94.12% vs 90.32%, *P* = 1.00) and blood loss (512.35 ± 727.00 ml vs 804.00 ± 838.98 ml, *P* = 0.23) between the artery embolization and non-embolization groups. Two patients in the caesarean section group required hysterectomy. |  |
| Liaquat et al[^70^](#_ENREF_70) | Pakistan | 2006 | Cohort | 5 | 49 | MToP | 14-26 weeks | 96.3% complete abortion within 48 hours  (83.3% required syntocinon and 14.8% required D&E); nil ruptures in the 5 women with previous CS |  |
| Limongelli et al[^141^](#_ENREF_141) | Italy | 2004 | Prospective non-randomised  controlled study | 120 | 462 | SToP | <13 weeks | Misoprostol ripening reduced need for  mechanical dilation with no cases of rupture in this study |  |
| Liu et al[^171^](#_ENREF_171) | China | 2003 | Case series | 4 | No | SToP | 8-9 weeks | 4 patients with undiagnosed CSP and major  haemorrhage at time of first trimester SToP; UAE successfully used in all cases to control haemorrhage with fertility preservation; one patient requested hysterectomy at follow up |  |
| Lohr et al[^197^](#_ENREF_205) | UK | 2018 | Retrospective cohort | 36 | 512 | SToP | 18-24 weeks | More than 1 previous CS significantly increased procedure time but no increase in complications; 1 case of major haemorrhage requiring hysterectomy and UAE for abnormal placentation; no cases of rupture |  |
| Long et al[^177^](#_ENREF_177) | China | 2022 | Retrospective cohort | 57 | 204 | MToP | Mid-trimester^‡^ | Placental location was strongly associated with higher risk of haemorrhage (P=0.001) or requiring emergency UAE +/- balloon tamponade. Prior caesarean was more common in women with placenta praevia than in the group with non-praevia. Previous CS was not an independent risk factor for haemorrhage. Paper suggests a cut off of 1.7cm for placental edge to os in diagnosing likelihood of haemorrhage with MToP (sensitivity 75.0%; specificity 86.7%) and suggest consideration of prophylactic UAE in these cases. |  |
| Lyus et al[^142^](#_ENREF_142) | UK | 2013 | Cohort | 21 | 253 | SToP | 18-22 weeks | No cases of uterine perforation, haemorrhage or inability to complete the procedure; no direct comparison between women with and without previous CS for other outcomes |  |
| Marinoni et al[^71^](#_ENREF_71) | Italy | 2007 | Retrospective cohort | 62 | 362 | MToP | 13-23 weeks | No difference in efficacy between women  with and without scar (98.4% vs 98.6%), no differences in complications including haemorrhage >500ml, transfusion or need for hysterotomy. One woman with scarred uterus required hysterectomy for bleeding. No cases of rupture. |  |
| Mark et al[^152^](#_ENREF_152) | USA | 2018 | Retrospective cohort | 637 | 1831 | SToP | First and second trimester^‡^ | No significant difference in adverse outcomes with SToP between women with and without previous CS (OR 1.33, CI 0.74-2.38) |  |
| Masse et al[^98^](#_ENREF_98) | USA | 2020 | Retrospective cohort | 51 | 417 | MToP | 14-24 weeks | Complication rates were comparable between people requiring more than or less than 5 doses of misoprostol for mid-trimester medical abortion. 1 case of rupture among 51 women with prior CS. |  |
| Mazouni et al[^206^](#_ENREF_206) | France | 2006 | Retrospective cohort | 50 | 202 | MToP | >15 weeks | No difference in failure rates or time to abortion between women with and without previous CS. Higher risk of retained placenta in group with previous CS (70% vs 50%, p=0.025), but no difference in haemorrhage (2% vs 0.9%, p=0.65). 2 cases of rupture in those with prior CS. |  |
| Meaidi et al[^100^](#_ENREF_100) | Denmark | 2019 | Retrospective cohort | 16912 | 69,525 | MToP | <9 weeks | Previous CS was a risk factor for need for  surgical intervention in women undergoing early MToP (OR 1.47, CI 1.33-1.62) |  |
| Meaidi et al^198^ | Denmark | 2020 | Retrospective cohort | 236 | 2532 | MToP | 13-23 weeks | Previous CS was not associated with need for surgical evacuation/RPOC after second-trimester MToP (OR 1.13, CI 0.89-1.43). 2 cases of rupture in those with prior CS. |  |
| Mentula et al[^131^](#_ENREF_131) | Finland | 2012 | Data analysis from  randomised controlled trial | 20 | 207 | MToP | 13-22 weeks | Previous CS was not associated with increased risk of need for surgical intervention (OR 1.23, CI 0.47-3.23). Rupture rate not reported. |  |
| Mobusher et al[^72^](#_ENREF_72) | Pakistan | 2013 | Cohort | 100 | 100 | MToP | 14-24 weeks | No significant differences in complication rates including haemorrhage, infection, retained placenta; no cases of uterine rupture |  |
| Morra et al[^73^](#_ENREF_73) | Italy | 2019 | Retrospective cohort | 340 | 1474 | MToP | 13-24 weeks | 9 uterine ruptures, all in women with previous CS and no difference in rupture risk with or without addition of mifepristone |  |
| Munir et al[^74^](#_ENREF_74) | Pakistan | 2014 | Randomised controlled trial | 150 | No | MToP | 13-26 weeks | Misoprostol was more efficacious than PGF2a  or Foley catheter with successful abortion in 82% (compared with 58% and 44% respectively) |  |
| Murphy et al[^149^](#_ENREF_149) | USA | 2012 | Retrospective cohort | 144 | 900 | SToP | 13-24 weeks | Previous CS was the only variable with  independent association with increased risk of major complication 6.3% vs 1.6% (OR 4.2, 1.79-9.93) |  |
| Naguib et al[^75^](#_ENREF_75) | Egypt | 2010 | Cohort | 50 | No | MToP | 16-26 weeks | 90% successful abortion; no cases or rupture;  3 women required hysterotomy: 1 for severe haemorrhage and 2 for failed abortion |  |
| Nair et al[^132^](#_ENREF_132) | UK | 2012 | Retrospective cohort | 25 | 651 | MToP | Unspecified^‡^ | No patients in either group had any  complications requiring admission |  |
| Obata-Yasuoka et al[^76^](#_ENREF_76) | Japan | 2009 | Retrospective cohort | 26 | 147 | MToP | 12-21 weeks | Non-significant difference in abortion success,  blood loss, dose required or blood transfusion; no cases of uterine rupture or failed abortion |  |
| Odeh et al^133^ | Israel | 2010 | Retrospective cohort | 16 | 387 | MToP | <7 weeks | Previous CS associated with higher risk of  need for surgical intervention (OR 2.39, CI 1.103-5.16), however so was previous vaginal delivery (OR 2.07, CI 1.11-3.86) |  |
| Ou et al[^172^](#_ENREF_172) | China | 2019 | Case series | 12 | 16 | Both | 13-28 weeks | All 28 women had uterine preservation; 8  with prenatal diagnosis all received prophylactic UAE followed by either hysterotomy or MToP; 20 cases diagnosed after delivery of the fetus and with adjuvant treatments including UAE, MTX or hysteroscopic resection of placenta |  |
| Patel et al[^143^](#_ENREF_143) | USA | 2006 | Retrospective descriptive | 123 | 1972 | SToP | 12-24 weeks | Overall misoprostol priming +/- laminaria was safe and effective; increased risk of adverse events^††^ in women with previous CS (48.8 per 1000 vs 16.2 per 1000; OR 3.11, CI 1.14-7.98; p<0.0087) |  |
| Peng et al[^77^](#_ENREF_77) | China | 2015 | Cohort | 32^§^ | No | MToP | 13-16 weeks | LUS thickness <3mm significantly associated  with uterine rupture during mid-trimester MToP (OR 94, CI 4.2-2106mm); interdelivery interval non-significant for increase in rupture |  |
| Petca et al[^101^](#_ENREF_101) | Romania | 2019 | Prospective cohort | 7 | 47 | MToP | 16-24 weeks | Mifepristone-misoprostol was associated with significantly shorter abortion time compared with misoprostol plus oxytocin regimen. No cases of uterine rupture. |  |
| Pongsatha et al[^78^](#_ENREF_78) | Thailand | 2011 | Retrospective descriptive | 64 | 677 | MToP | 14-32 weeks | No ruptures out of 64 women with  previous CS |  |
| Pongsatha et al[^102^](#_ENREF_102) | Thailand | 2024 | Prospective comparative | 80 | 158 | MToP | 14-28 weeks | Successful MToP was not significantly different between those with and without prior CS. Also non-significant between the groups were haemorrhage, need for surgical intervention, abortion time. 1 case of rupture in woman with 1 previous CS. |  |
| Pourhoseini et al^103^ | Iran | 2024 | Randomised controlled trial | 104 | 104 | MToP | <20 weeks | Adjuvant letrozole reduced abortion time and increased complete abortion with misoprostol medical termination of pregnancy. No ruptures reported. |  |
| Pridmore et al[^150^](#_ENREF_150) | Australia | 1999 | Case series |  |  | SToP | 4-20 weeks | Previous CS not a risk factor for perforation  in the first trimester; in the first 4 years of the study, previous CS associated with 19-fold increase in perforation risk (0.26% (3/1155 women with no previous CS vs 5% (3 in 60 women with previous CS)); overall reduction in perforation rates from 0.13% to 0.02% with introduction of protocols recommending prostaglandin and osmotic dilators for women with previous gynaecological surgery |  |
| Ramesh et al[^144^](#_ENREF_144) | USA | 2014 | Retrospective cohort | 29 | 114 | SToP | 14-16 weeks | Procedure time, bleeding and pain experience not different between misoprostol and osmotic dilator groups; women with previous CS had longer procedure time (p=0.029) |  |
| Rashbaum et al[^173^](#_ENREF_173) | USA | 1995 | Case series | 7 | N/A | SToP | 13-23 weeks | 7 cases of placenta accreta encountered  amongst 16,827 (0.04%) second-trimester D&E for abortion; all 7 had history of one or more previous CS and 5/7 had ultrasound showing placenta praevia; all required hysterectomy to control bleeding |  |
| Reehan et al[^104^](#_ENREF_104) | Iraq | 2024 | Cross-sectional | 100 | N/A | MToP | 13-26 weeks | Increasing number of prior CS associated with longer abortion time (P=0.01). 2 cases of rupture, both in women with 3 prior CS using low dose 100-200mcg PV misoprostol |  |
| Reeves et al^134^ | USA | 2016 | Pooled data analysis  from randomised controlled trial | 238 | 1921 | MToP | <9 weeks | Prior CS not associated with increased risk  of need for surgical uterine evacuation |  |
| Reischer et al[^112^](#_ENREF_112) | Austria | 2023 | Retrospective cohort | 139 | 774 | MToP | 14-24 weeks | Prior CS was not associated with increased abortion time. No cases of rupture. |  |
| Schneider et al[^151^](#_ENREF_151) | Israel | 1994 | Retrospective cohort | 37^‡‡^ | 996 | SToP | 14-22 weeks | No cases of perforation or rupture. Eight  cases of DIC all in women without previous CS. Previous CS not associated with prolonged procedure time or severe complication for women having induced abortion with D&C |  |
| Scioscia et al[^79^](#_ENREF_79) | Italy | 2005 | Retrospective cohort | 63^§§^ | 142 | MToP | 13-23 weeks | No significant differences in complications  (p=0.78), heavy bleeding (p=0.33), transfusion (p=0.31) or failed abortion (p=0.55); no cases of rupture |  |
| Shammas et al[^80^](#_ENREF_80) | Jordan | 2006 | Retrospective cohort | 63 | 457 | MToP | 15-28 weeks | No difference in abortion time or dose  required, increased risks of incomplete abortion for women with previous CS (82% vs 60%) and EBL >500ml (11.5% vs 6.1%). No cases of rupture and no increase in severe complications. |  |
| Shantikumar et al[^105^](#_ENREF_105) | India | 2021 | Cohort | 20 | 180 | MToP | 13-20 weeks | Two sequential doses of mifepristone prior to misoprostol reduced abortion time. No cases of rupture. |  |
| Sharma et al[^113^](#_ENREF_113) | Nepal | 2020 | Retrospective descriptive | 6 | 34 | MToP | 13-28 weeks | Mifepristone and misoprostol are safe and effective for second-trimester medical abortion. No cases of rupture. |  |
| Shay et al[^106^](#_ENREF_106) | USA | 2022 | Retrospective cohort | 15 | 79 | MToP | 14-28 weeks | Mifepristone and misoprostol lead to shorter abortion time compared to misoprostol alone. No cases of rupture. |  |
| Spingler et al^199^ | Germany | 2023 | Retrospective cohort | 77 | 339 | Both | All trimester (median 17.4 weeks)^‡ ¶¶^ | Prior CS did not increase risk of complications across all procedures^§§^. All women with prior CS undergoing MToP used oral mifepristone followed by dinoprostone and were not used in analysis of rupture rates for this reason. |  |
| Stewart et al[^107^](#_ENREF_107) | Australia | 2022 | Retrospective cohort | 75 | 332 | MToP | ≥20 weeks (average 23 weeks; IQR 22-26)^‡^ | 2 cases of uterine rupture occurred: both in women with a single prior CS. The hospital adopted a low-dose misoprostol regimen following these cases. |  |
| Tarim et al[^81^](#_ENREF_81) | Turkey | 2005 | Retrospective cohort | 12 | 45 | MToP | Second trimester^‡^ | No cases of uterine rupture; no significant  differences in abortion time, hospital stay or haemoglobin levels after delivery |  |
| Torriente et al[^82^](#_ENREF_82) | South Africa | 2017 | Retrospective cohort | 268 | 265 | MToP | 13-20 weeks | Women with previous CS more likely to have  retained placenta p<0.001, no uterine ruptures |  |
| Turgut et al[^83^](#_ENREF_83) | Turkey | 2013 | Retrospective cohort | 56 | 163 | MToP | 13-24 weeks | No differences in failed MToP (p=0.056) or  blood transfusion (p=0.05). Laparotomy (p=0.004), uterine rupture(p=0.016) and hysterotomy (P<0.001) more frequent in women with previous CS compared to women without. Abortion time lower in women without previous CS (p=0.031) |  |
| Uribe et al[^118^](#_ENREF_118) | USA | 2019 | Retrospective cohort | 29 | 96 | MToP | 14-23 weeks | No significant differences in haemorrhage,  transfusion or retained placenta between women with and without prior CS, including when analysed for women with more than one prior CS |  |
| van Beekhuizen et al[^174^](#_ENREF_174) | Finland | 2021 | Cohort | 7^†††^ |  | Both | 14-24 weeks | This paper describes 9 women who had  midtrimester abortion for accreta but little data on outcomes for these women, both MToP and hysterotomy were used as methods of abortion |  |
| van Bogaert [^84^](#_ENREF_84) | South Africa | 2007 | Retrospective cohort | 48 | 639 | MToP | <20 weeks | No significant difference in need for repeat  misoprostol (p=0.11) or surgical intervention (p=0.77) |  |
| Velipasaoglu et al[^85^](#_ENREF_85) | Turkey | 2018 | Prospective cohort | 104 | 233 | MToP | 14-22 weeks | No significant differences in abortion time,  retained placenta, blood transfusion; no cases of uterine rupture or major complication |  |
| Vlad et al[^108^](#_ENREF_108) | Canada | 2022 | Retrospective cohort | 9 | 50 | MToP | 13-24 weeks | No cases of rupture. |  |
| Wagner et al[^126^](#_ENREF_126) | Germany | 2011 | Retrospective cohort | 26 | 158 | MToP | 11-24 weeks | Previous CS was associated with increased  abortion time compared with women with no previous CS (p<0.0001)^ƗƗ^ |  |
| Wang et al[^44^](#_ENREF_44) | China | 2010 | Cohort | 668 | No | MToP | <7 weeks | No cases of rupture or major complications  reported |  |
| Wilson et al[^153^](#_ENREF_153) | USA | 2011 | Retrospective cohort | 71 | 516 | SToP | 12-18 weeks | None of the women with previous CS had surgical complications. |  |
| Xie et al[^175^](#_ENREF_175) | China | 2017 | Case series | 12^‡‡‡^ | No | MToP | 15-28 weeks | 6 patients recived UAE prior to hysterotomy  or MToP resulting in less blood loss (average 383ml with UAE and 1533ml without UAE) and reduction in hospital stay (9 days vs 18 days). No cases required hysterectomy. |  |
| Xu et al[^45^](#_ENREF_45) | China | 2001 | Prospective cohort | 35 | 157 | MToP | <7 weeks | No differences in rates of complete abortion  or abortion time between women with and without previous CS; no cases of rupture or major complications reported |  |
| Young et al[^46^](#_ENREF_46) | USA | 2022 | Retrospective cohort | 950 | 7875 | MToP | <10 weeks | Buccal administration of misoprostol is associated with a higher proportion of complete abortion before 64 days of gestation compared to vaginal misoprostol. Clinically, vaginal misoprostol is an effective route of administration through 70 days of gestation. No direct comparison between participants with and without CS. No cases or rupture. |  |
| *CS, Caesarean section; MToP, medical termination of pregnancy; SToP, surgical termination of pregnancy; USA, United States of America; OR, odds ratio; CI, confidence interval; CSD Caesarean scar defect; ToP, termination of pregnancy; EBL estimated blood loss; UAE uterine artery embolisation; DIC disseminated intravascular coagulation; D&E, dilation and evacuation; HCG, human chorionic gonadotrophin; UK, United Kingdom; CSP caesarean scar pregnancy; RPOC, retained products of conception; PGF2a, prostaglandin F2a; MTX methotrexate; LUS lower uterine segment; cc, cubic centimetres; ml, millilitres*  ^†^ Included women with scarring from myomectomy with no breakdown of numbers  ^‡^ Gestational age range not specified  ^§^ MToP performed in 38% and SToP performed in 62%  ^¶^ One excluded due to prior uterine surgery not CS; not able to be excluded for efficacy analysis  ^††^ Adverse events included: fever >38.0 degrees celcius; anaphylaxis; cervical laceration; uterine rupture; hysterectomy; death; spontaneous rupture of membranes prior to D&E; delivery of fetus prior to D&E; haemorrahge>500ml; hospital admission  ^‡‡^The other 33 women with previous CS had missed abortions  ^§§^ Including 2 women with previous transmural myomectomy  ^¶¶^ Did not separate outcomes by women with and without CS by trimester or mode of termination; unable to extract data solely on SToP  ^†††^at least 7 of 9 women who had ToP had accreta overlying scar suggesting previous CS  ^‡‡‡^ A majority of women had had previous CS but exact number unclear | | | | | | | | | |
